# Phonon-induced disorder in dynamics of optically pumped metals from nonlinear electron-phonon coupling

**DOI:** 10.1038/s41467-021-26030-3

**Published:** 2021-10-04

**Authors:** John Sous, Benedikt Kloss, Dante M. Kennes, David R. Reichman, Andrew J. Millis

**Affiliations:** 1 Department of Physics, Columbia University, New York, NY 10027 USA; 2 Department of Chemistry, Columbia University, New York, NY 10027 USA; 3 JARA - Fundamentals of Future Information Technology, Institut für Theorie der Statistischen Physik, RWTH Aachen, 52056 Aachen, Germany; 4 Max Planck Institute for the Structure and Dynamics of Matter and Center for Free-Electron Laser Science, 22761 Hamburg, Germany; 5 Center for Computational Quantum Physics, Flatiron Institute, 162 5th Avenue, New York, NY 10010 USA

**Keywords:** Electronic properties and materials, Superconducting properties and materials, Thermodynamics

## Abstract

The non-equilibrium dynamics of matter excited by light may produce electronic phases, such as laser-induced high-transition-temperature superconductivity, that do not exist in equilibrium. Here we simulate the dynamics of a metal driven at initial time by a spatially uniform pump that excites dipole-active vibrational modes which couple nonlinearly to electrons. We provide evidence for rapid loss of spatial coherence, leading to emergent effective disorder in the dynamics, which arises in a system unitarily evolving under a translation-invariant Hamiltonian, and dominates the electronic behavior as the system evolves towards a correlated electron-phonon long-time state, possibly explaining why transient superconductivity is not observed. Our framework provides a basis within which to understand correlation dynamics in current pump-probe experiments of vibrationally coupled electrons, highlight the importance of the evolution of phase coherence, and demonstrate that pumped electron-phonon systems provide a means of realizing dynamically induced disorder in translation-invariant systems.

## Abstract (web summary)

Superconductivity reported in metals driven away from equilibrium via optical pumping has been proposed to arise from nonlinear coupling between electrons and optically excited phonons. The authors use an exact approach to show that here, disorder, which disfavors superconductivity, emerges even though the system is translationally invariant.

## Introduction

Major efforts in condensed-matter physics are currently focused on the means to induce novel phases of matter and harness their properties for practical gain. For many years such phases were thought to robustly exist only as equilibrium, thermodynamic states. The potential out-of-equilibrium induction of transient phases, enabled by recent experimental advances in the creation and utilization of tailored time-resolved external fields that can excite specific degrees of freedom, opens a door to new modalities for the realization and control of new electronic states^1,2^.

Optical, mode-specific excitation of atomic vibrations^3^ serves as one broad class of out-of-equilibrium techniques that has been shown experimentally to lead to dramatic modifications in electronic behavior^4–6^, including the possible induction of a superconducting transition at a critical temperature larger than its equilibrium counterpart in K_3_C_60_^7^, YBa_2_Cu_3_O_6.5_^8^, and organic salts^9^. In general, optically accessible phonons are long-wavelength dipole-active modes, which typically do not couple linearly to the electron density, and therefore nonlinearities are expected to govern the dynamics in centrosymmetric systems^10–13^, stimulating many interesting theoretical proposals^13–20^. One particular mechanism^13^ is based on the observation that since direct, local interaction between electrons and photo-excited phonons must depart from that of conventional linear Holstein^21^ and Fröhlich^22,23^ models, one must consider a quadratic coupling of driven phonons to the electron density. An approximate analysis of such a model was presented previously^13^ (see Supplementary Note 3). Here, we use exact numerical methods and an effective theory based on a low-order expansion in the electron–phonon coupling to unravel the emergent electronic behavior in this driven, nonequilibrium system. Combining a tensor-network approach for the time evolution of an infinite one-dimensional (1D) system on short timescales with propagation to long times using direct Krylov subspace methods for finite-size systems and analytical arguments, we elucidate the spatially resolved dynamics of electrons coupled to pumped phonons. Our main results are:

(1) *Phonon-induced disorder*: we observe fast growth of local electronic correlations after the application of the pump. A dramatic flattening in the momentum dependence of charge, spin, and pairing correlations rapidly follows, pointing to the loss of electronic spatial phase coherence. We find that disorder emerges as a result of the nature of the initial light-created coherent phonon superposition state whose dynamics is effectively governed by a Hamiltonian that approximately conserves phonon occupations. The presence of quasi-conserved phonon constants of motion implies that electronic observables self-average over the different disordered phonon configurations of the initial state and possess no off-diagonal coherence between different phonon sectors. This provides a realization of disorder-free localization^24–26^, recently discussed in the context of lattice gauge theories^27–31^. To understand this behavior, we derive an effective model whose behavior captures the qualitative features of the exact dynamics on transient timescales. Our effective theory provides a natural framework within which disorder and electron localization arise in the dynamics, and provides a perspective for the short-time dynamics complementing analysis of the long-time behavior where the loss of phonon coherence and preservation of the Poisson-distributed diagonal eigenvalues of the initial coherent state density matrix characterizes a random disorder potential, responsible for the destruction of phase coherence of the normal state electronic correlations^13^.

(2) *Correlated electron–phonon steady state*: we provide evidence that the system evolves at long times to a steady state characterized by sizeable correlations between electrons and phonons. The early-time dynamics that follow the pump already indicate rapid growth of local, negative correlations between the electron density $\hat{n}$ and the oscillator quadratic displacement ${\hat{X}}^{2}$ at a given site and between the linear displacement $\hat{X}$ at adjacent sites, which signals a tendency towards charge flow between neighboring sites, resulting in enhanced double occupancy. This dynamical process quenches the Friedel oscillations^32^ of the electron density profile, and manifests as a space-time-dependent feature in the density-density correlation function that spreads spatially outwards along a “light cone” defined by the Fermi velocity^33^. Behind the light cone, very rapidly, the density–density correlation function becomes basically structureless, suggesting that the asymptotic state possesses a large degree of randomness. At long times, we find an overall increase in the magnitude of the expectation value of the electron–phonon interaction term, implying evolution towards a strongly correlated long-time electron–phonon state.

(3) *Dynamically induced strong-coupling behavior*: we compare the dynamical electronic behavior in response to a pump in the quadratic-coupling model against that in the linear (Holstein) counterpart. We observe larger double occupancy and greater large-amplitude response of momentum-resolved correlation peaks in the quadratic model, indicating that in this model, in contrast to the more widely studied Holstein model, the drive pushes the system into a strong-coupling regime. This substantive dynamical response of the nonlinearly coupled system implies the existence of nonequilibrium pathways to coherent induction of electronic phases not accessible in equilibrium, and highlights the importance of the quadratic coupling in irradiated materials.

### Physical setup

We consider a metal whose vibrational modes are excited at an initial time by a short-duration light pulse that creates a coherent phonon field^34^ on every site, which couples nonlinearly to the local electron density.

The Hamiltonian that governs the dynamics is given by

1 $${{{{{{{\mathcal{H}}}}}}}}={{{{{{{{\mathcal{H}}}}}}}}}_{{{{{{{{\rm{e}}}}}}}}}+{{{{{{{{\mathcal{H}}}}}}}}}_{{{{{{{{\rm{ph}}}}}}}}}+{{{{{{{{\mathcal{V}}}}}}}}}_{{{{{{{{\rm{e}}}}}}}}{\mbox{-}}{{{{{{{\rm{ph}}}}}}}}}.$$

Here ${{{{{{{{\mathcal{H}}}}}}}}}_{{{{{{{{\rm{e}}}}}}}}}=-J{\sum }_{i,\sigma }{c}_{i,\sigma }^{{{{\dagger}}} }{c}_{i+1,\sigma }+{{{{{{{\rm{h.c.}}}}}}}}$ characterizes the dynamics of electrons of spin flavor *σ* ∈ {*↑*, *↓*} via the fermion creation (annihilation) operator ${c}_{i,\sigma }^{{{{\dagger}}} }$ (*c*_*i*,*σ*_) and charge density ${\hat{n}}_{i}={\sum }_{\sigma }{\hat{n}}_{i,\sigma }$ at site *i*. The phonon Hamiltonian is ${{{{{{{{\mathcal{H}}}}}}}}}_{{{{{{{{\rm{ph}}}}}}}}}=\omega {\sum }_{i}\left({b}_{i}^{{{{\dagger}}} }{b}_{i}+\frac{1}{2}\right)$, which characterizes a local optical Einstein phonon mode with frequency *ω*(*ℏ* = 1), described by the boson creation (annihilation) operator ${b}_{i}^{{{{\dagger}}} }$ (*b*_*i*_). The electrons of this irradiated system couple locally to the excited vibrations via the the dominant symmetry-allowed interaction^13^

2 $${{{{{{{{\mathcal{V}}}}}}}}}_{{{{{{{{\rm{e}}}}}}}}{\mbox{-}}{{{{{{{\rm{ph}}}}}}}}}={g}_{q}\mathop{\sum}\limits_{i}({\hat{n}}_{i}-1){({b}_{i}^{{{{\dagger}}} }+{b}_{i})}^{2}.$$

This coupling also serves as a description of double-well ^35,36^ and other^37^ systems for which the linear approximation is inadequate. This model Hamiltonian implies an equilibrium renormalization of the oscillator stiffness $K\to K[1+4\frac{{g}_{q}}{\omega }({\langle \hat{n}\rangle }_{i}-1)]$. Thus, the onsite harmonic oscillator is stable so long as $\left|{g}_{q}\right| < \frac{\omega }{4}$^13^ (see Supplementary Note 1).

Since the wavelength of the pump field extends beyond the lattice scale, we assume it produces a perfectly phase-coherent initial product state of onsite phonon coherent states ${\bigotimes }_{i}{\left|\alpha \right\rangle }_{i}$, where $\left|\alpha \right\rangle ={e}^{-\frac{{\left|\alpha \right|}^{2}}{2}}{\sum }_{\nu }\frac{{\alpha }^{\nu }}{\sqrt{\nu !}}\left|\nu \right\rangle$ represents a coherent state of amplitude *α* written here as an appropriate superposition of phonon-number states $\left|\nu \right\rangle$. We simulate the time evolution of the initial state

3 $$\left|{{{{{{{\mathbf{\Psi }}}}}}}}\right\rangle =\left|{{{{{{{\rm{FS}}}}}}}}\right\rangle \otimes \mathop{\bigotimes}\limits_{i}{\left|\alpha \right\rangle }_{i},$$

where $\left|{{{{{{{\rm{FS}}}}}}}}\right\rangle ={\prod }_{k\le {k}_{{{{{{{{\rm{F}}}}}}}}}}{c}_{k,\uparrow }^{{{{\dagger}}} }{c}_{k,\downarrow }^{{{{\dagger}}} }\left|0\right\rangle$ with *k*_F_ = *π*/2 describes a metal formed from a Fermi sea of spinful electrons in a half-filled ($\langle {\hat{n}}_{i}\rangle =1$) 1D lattice.

Here, we set the lattice constant *a* = 1 and study the physics of the model for physical parameters defined in units of *J*, i.e., we set *J* = 1. In the main text, we consider *g*_*q*_ ≤ 0.25 for *ω* = *π*/2 to study dynamics of the nonlinear model for couplings ranging from weak to strong, and use $\alpha =\sqrt{2}$ for the pump amplitude. This choice of *ω* allows us to numerically resolve the quantum effects in dynamics due to a large yet amenable phonon Hilbert space.

We simulate the time evolution of $\left|{{{{{{{\mathbf{\Psi }}}}}}}}\right\rangle$ in an infinite system on transient timescales via the infinite time-evolved block decimation (iTEBD) algorithm^38^ and access its long-time behavior in finite-size systems of size *L* = 3–6 and local phonon Hilbert space dimension *d*_*ν*_ = 8, 10, 12 using direct Krylov subspace methods. In iTEBD, one employs a matrix-product state (MPS) ansatz for quantum states in the thermodynamic (infinite-size) limit, which permits access to information pertaining to long-range correlations in the system. Time evolution of an MPS is however ultimately limited to finite times because of the exponential growth of entanglement. Krylov subspace methods, based on Hamiltonian matrix-state vector multiplication, are in contrast not limited to short times, but are restricted to small *L* due to the exponential growth of the Hilbert space with *L*. Combining the two approaches allows us to derive reliable conclusions about long-range correlations on finite timescales from iTEBD and local correlations at long times from Krylov propagation.

## Results

**Fig. 1:**
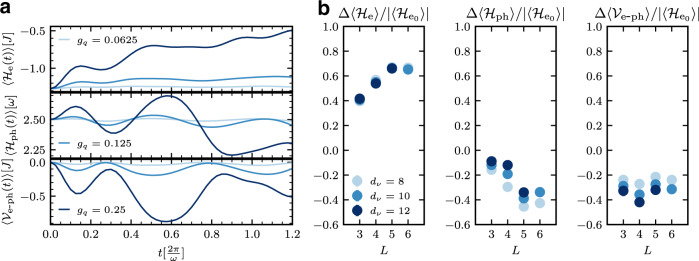
Energy redistribution among the different system subsectors. **a** Infinite system iTEBD simulations of the time dependence of the electronic (top), phononic (middle) and electron-phonon (bottom) energy densities for *ω* = *π*/2 show a trend with larger *g*_*q*_ of rapid heating of the electronic subsector, accompanied by transient relaxation of the electron-phonon subsector. **b** Exact Krylov propagation of small systems with *L* = 3 − 6 and local phonon Hilbert space dimension *d*_*ν*_ = 8, 10, 12 (*L* = 6 is restricted to *d*_*ν*_ = 10) for *ω* = *π*/2 and the largest coupling strength *g*_*q*_ = 0.25 to asymptotically long times showing the net change relative to the initial state in electronic (left), phononic (center) and electron-phonon (right) energy densities confirms a correlated electron-phonon steady state, as evidenced by the considerable flow of energy from the electron-phonon subsector to the electronic subsector. The y-axis labels of the net change in energy densities have been placed at the top of the corresponding plots. Here, ${{{{{{{{\mathcal{H}}}}}}}}}_{{{{{{{{{\rm{e}}}}}}}}}_{0}}\equiv {{{{{{{{\mathcal{H}}}}}}}}}_{{{{{{{{\rm{e}}}}}}}}}(0)$.

Figure 1 demonstrates the energy redistribution amongst the different system subsectors in the course of the time evolution on timescales ranging from short (panel a) to long (panel b), as the system approaches its long-time limit of a correlated electron–phonon steady state. Consider the largest coupling *g*_*q*_ = 0.25 (dark lines in panel a). At early times $t\le \frac{2\pi }{\omega }$, the electron subsystem absorbs energy from the excited phonons, and the phonon energy density oscillates about a value close to its initial value, while the electron–phonon energy density becomes more negative, see panel a of Fig. 1. At asymptotically long times, we observe an overall flow of energy from the phonon and electron–phonon subsectors to the electron subsector (panel of b of Fig. 1). Importantly, the increase in the magnitude of the (negative) electron–phonon correlation term implies a long-time correlated electron–phonon state.

**Fig. 2:**
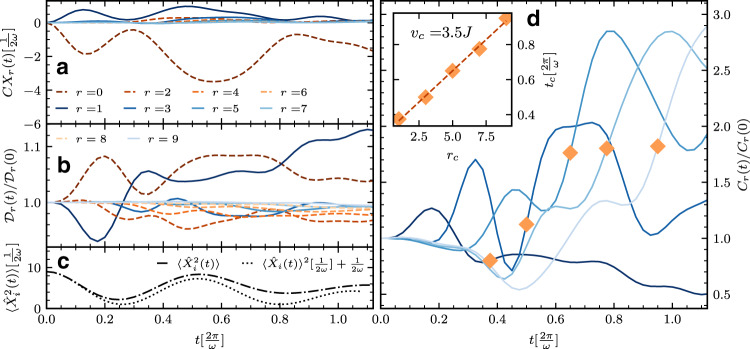
Dynamics of charge and charge-phonon correlations. **a**–**c** Time evolution of charge–lattice correlation $C{X}_{r}(t)=\langle {\hat{n}}_{i}{\hat{X}}_{i+r}^{2}(t)\rangle -\langle {\hat{n}}_{i}(t)\rangle \langle {\hat{X}}_{i+r}^{2}(t)\rangle$ (**a**) contrasted against that of $\langle {\hat{X}}_{i}^{2}(t)\rangle$ (**c**), and of the density–density correlation ${{{{{{{{\mathcal{D}}}}}}}}}_{r}(t)=\langle {\hat{n}}_{i}{\hat{n}}_{i+r}(t)\rangle$ normalized with respect to its initial-time value ${{{{{{{{\mathcal{D}}}}}}}}}_{r}(0)$ (**b**). Here, ${\hat{X}}_{i}:= \sqrt{\frac{1}{2M\omega }}({b}_{i}^{{{{\dagger}}} }+{b}_{i})$, where *M* is the oscillator mass, which we set to unity, *M* = 1. Note the violation of the relation ${({{\Delta }}{X}_{i}(t))}^{2}=\langle {\hat{X}}_{i}^{2}(t)\rangle -{\langle {\hat{X}}_{i}(t)\rangle }^{2}=\frac{1}{2\omega }$ for *t* ⪆ $0.15\frac{2\pi }{\omega }$, an indication of deviation of the oscillator from an ideal coherent state. **d** Onset of a light-cone profile in the normalized density–density charge correlations; here, ${C}_{r}(t)=\langle {\hat{n}}_{i}{\hat{n}}_{j}(t)\rangle -\langle {\hat{n}}_{i}(t)\rangle \langle {\hat{n}}_{j}(t)\rangle$ is normalized with respect to its initial-time metallic Friedel oscillations profile *C*_*r*_(0). The diamond symbols mark the inflection point preceding the second maximum for the different *r* lines, which we use in the inset to find the best fit of the light-cone charge propagation *t*_c_ versus *r*_c_ (dashed line), yielding an estimate for charge velocity: *v*_c_ ≈ 3.5*J*. We use *g*_*q*_ = 0.25 and *ω* = *π*/2 in this figure.

Correlations between electrons and phonons already appear in the early-time dynamics, as we demonstrate in Fig. 2. Consider the charge–phonon correlation function $C{X}_{r}(t)=\langle {\hat{n}}_{i}{\hat{X}}_{i+r}^{2}(t)\rangle -\langle {\hat{n}}_{i}(t)\rangle \langle {\hat{X}}_{i+r}^{2}(t)\rangle$ (Fig. 2, panel a), where ${\hat{X}}_{i}:= \sqrt{\frac{1}{2M\omega }}({b}_{i}^{{{{\dagger}}} }+{b}_{i})$, and we set *M* = 1. For *r* = 0, $\hat{n}$ rapidly becomes negatively correlated with ${\hat{X}}^{2}$. Note that $\langle {\hat{n}}_{i}(t)\rangle =1$ throughout the dynamics in the translationally invariant system under consideration and $\langle {\hat{X}}_{i}^{2}(t)\rangle$ (dash-dotted line, panel c) remains positive under time evolution. The substantial local, negative correlations in *CX*_0_(*t*), therefore, imply a flow of electrons between neighboring sites. The same analysis applied to *CX*_1_(*t*) reveals a positive correlation between electron density and phonons separated by a single site with a dynamical profile somewhat similar (albeit of opposite sign) to *CX*_0_(*t*). With a slightly delayed onset, much weaker positive correlations build-up at larger *r* in *CX*_*r*_(*t*). The interplay between onsite and nearest-neighbor correlations in *CX*_*r*_(*t*) reflects the tendency of charge to flow from a site to its neighbors, implying that doublons (doubly occupied sites) and holons (empty sites) emerge in the dynamics on such timescales. Indeed, in panel b, we observe a rapid enhancement of local electron density–density correlations ${{{{{{{{\mathcal{D}}}}}}}}}_{0}(t)=\langle {\hat{n}}_{i}{\hat{n}}_{i}(t)\rangle =\langle {\hat{n}}_{i}\rangle +2\langle {\hat{n}}_{i,\uparrow }{\hat{n}}_{i,\downarrow }(t)\rangle$, accompanied by the suppression of ${{{{{{{{\mathcal{D}}}}}}}}}_{1}(t)=\langle {\hat{n}}_{i}{\hat{n}}_{i+1}(t)\rangle$ due to doublon creation, as expected if there is a tendency towards formation of an alternating pattern of doubly and singly occupied sites. For times greater than $t\approx 0.175[\frac{2\pi }{\omega }]$, ${{{{{{{{\mathcal{D}}}}}}}}}_{1}(t)$ begins to grow and becomes positive, whilst ${{{{{{{{\mathcal{D}}}}}}}}}_{2}(t)=\langle {\hat{n}}_{i}{\hat{n}}_{i+2}(t)\rangle$ diminishes, and a wavefront behavior in *r* appears to arise. In fact, when normalized against the *t* = 0 metal Friedel density profile, a density–density correlation light-cone *C*_*r*_(*t*)/*C*_0_(*t*) (${C}_{r}(t)=\langle {\hat{n}}_{i}{\hat{n}}_{j}(t)\rangle -\langle {\hat{n}}_{i}(t)\rangle \langle {\hat{n}}_{j}(t)\rangle$)^33,39^ propagating outwards in *r* can be clearly seen (Fig. 2, panel d). A characteristic feature that emerges for larger *r* at later time delays closely trails the second-in-time maximum. Thus, to sharply characterize the light cone, we track the inflection point preceding that maximum (diamond symbols). A line of best fit through these data points (Fig. 2, panel d; inset) reveals linear charge propagation with a velocity *v*_c_ ≈ 3.5*J*, slightly larger than the free metal Fermi velocity 2*k*_F_*J* = *πJ*. On the timescales accessed by iTEBD, we find no evidence for a wavefront propagating in either of *CX*_*r*_(*t*) or $\langle {\hat{X}}_{i}(t){\hat{X}}_{i+r}(t)\rangle$, reflecting the resistance to the propagation of the dispersionless Einstein oscillator modes of the initial-time (*g*_*q*_ = 0) state. The behavior exhibited by *CX*_*r*_(*t*) and *C*_*r*_(*t*) implies nonequilibrium induction of enhanced double occupancy 〈*n*_*i*,*↑*_*n*_*i*,*↓*_(*t*)〉, which we have directly verified.

**Fig. 3:**
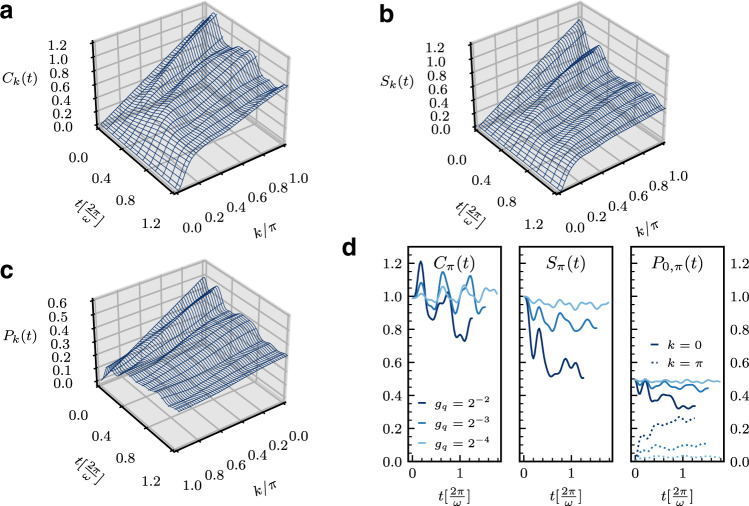
Dynamics of momentum-resolved electronic correlations. We study the evolution with time of momentum-resolved charge ${C}_{k}(t)={{{{{{{\mathcal{F}}}}}}}}\{{C}_{r}(t)\}$ (**a**), spin ${S}_{k}(t)={{{{{{{\mathcal{F}}}}}}}}\{{S}_{r}(t)\}$ (**b**), and pairing ${P}_{k}(t)={{{{{{{\mathcal{F}}}}}}}}\{{P}_{r}(t)\}$ (**c**) correlation functions for *g*_*q*_ = 0.25 and *ω* = *π*/2 and the dependence on time of certain *k* (0, *π*) correlations for various *g*_*q*_ at *ω* = *π*/2 (**d**). Note the *k*-axis of the *P*_*k*_(*t*) plot in (**c**) has been inverted for better visibility, and the *y*-axis labels of the 0/*π* correlations in (**d**) have been placed at the top of the corresponding plots. Here, ${C}_{r}\equiv \langle {\hat{n}}_{i}{\hat{n}}_{i+r}\rangle -\langle {\hat{n}}_{i}\rangle \langle {\hat{n}}_{i+r}\rangle ,{S}_{r}\equiv \langle ({\hat{n}}_{i,\uparrow }-{\hat{n}}_{i,\downarrow })({\hat{n}}_{i+r,\uparrow }-{\hat{n}}_{i+r,\downarrow })\rangle$ and ${P}_{r}\equiv \langle {c}_{i,\uparrow }^{{{{\dagger}}} }{c}_{i,\downarrow }^{{{{\dagger}}} }{c}_{i+r,\downarrow }{c}_{i+r,\uparrow }\rangle$. ${{{{{{{\mathcal{F}}}}}}}}$ denotes the Fourier transform. Charge, spin, and pairing correlations all rapidly flatten in the course of the dynamics. Note conservation of *C*_0_(*t*) and *S*_0_(*t*) in the dynamics.

Turning to the dynamics of long-range electronic correlations, in Fig. 3 we study the evolution with time of the momentum-resolved charge *C*_*k*_(*t*), spin *S*_*k*_(*t*), and pairing *P*_*k*_(*t*) correlation functions to fully characterize the electronic features. Apart from the fast initial growth of *C*_*π*_(*t*) for *t* ⪅ $0.2[\frac{2\pi }{\omega }]$ due to the enhanced double occupancy, we observe rapid flattening in momentum space of these correlations, marking the loss of spatial coherence, despite the persistent growth of local density–density and charge–phonon correlations, indicating that the pattern of doubly and singly occupied sites is becoming random. This remarkable behavior implies an effective disordered state forms on transient timescales, and a more subtle role played by phonons in the dynamics, as we show next.

### Effective model for the disorder

To understand the mechanism behind the appearance of disorder, we derive an effective theoretical picture for the dynamics to leading order in *g*_*q*_/*ω*.

We find it convenient to consider a rotating frame in which the off-diagonal phonon terms (in the occupation-number basis) of Eq. (2) are eliminated via a Bogoliubov-type squeezing transformation^13^: ${{{{{{{\mathcal{H}}}}}}}}\to \tilde{{{{{{{{\mathcal{H}}}}}}}}}=U{{{{{{{\mathcal{H}}}}}}}}{U}^{{{{\dagger}}} }$, where *U* = *e*^*S*^, $S=-{\sum }_{j}\frac{1}{2}{\zeta }_{j}({b}_{j}^{{{{\dagger}}} }{b}_{j}^{{{{\dagger}}} }-{b}_{j}{b}_{j})$, and ${\zeta }_{i}=-\frac{1}{4}{{{{{{\mathrm{ln}}}}}}}\,[1+4\frac{{g}_{q}}{\omega }({\hat{n}}_{i}-1)]$, the squeezing parameter, is chosen so that the ${({b}_{i}^{{{{\dagger}}} })}^{2}$ and ${({b}_{i})}^{2}$ terms vanish. This yields, in the squeezed frame, $\tilde{{{{{{{{\mathcal{H}}}}}}}}}={e}^{S}{{{{{{{{\mathcal{H}}}}}}}}}_{{{{{{{{\rm{e}}}}}}}}}{e}^{-S}+{\sum }_{i}\omega \sqrt{1+4\frac{{g}_{q}}{\omega }({\hat{n}}_{i}-1)}({\beta }_{i}^{{{{\dagger}}} }{\beta }_{i}+\frac{1}{2})$, where *β*^†^ creates a squeezed-phonon state. Perturbatively expanding the transformed coupling term in orders of *g*_*q*_/*ω*, we find

4 $${{{{{{{\mathcal{{H}}}}}}}_{{{{{{{{\rm{eff.}}}}}}}}}}}	= -{J}^{* }\mathop{\sum}\limits_{i,\sigma }({c}_{i,\sigma }^{{{{\dagger}}} }{c}_{i+1,\sigma }+{{{{{{{\rm{h.c.}}}}}}}})+{\omega }^{* }\mathop{\sum}\limits_{i}\left({\beta }_{i}^{{{{\dagger}}} }{\beta }_{i}+\frac{1}{2}\right)\\ 	\quad+2{g}_{q}\mathop{\sum}\limits_{i}({\hat{n}}_{i}-1)\left({\beta }_{i}^{{{{\dagger}}} }{\beta }_{i}+\frac{1}{2}\right)\\ 	\quad-4\frac{{g}_{q}^{2}}{\omega }\mathop{\sum}\limits_{i}({\hat{n}}_{i,\uparrow }-1/2)({\hat{n}}_{i,\downarrow }-1/2)\left({\beta }_{i}^{{{{\dagger}}} }{\beta }_{i}+\frac{1}{2}\right).$$

Here, ${J}^{* }=J{e}^{-\frac{1}{2}{(\frac{{g}_{q}}{\omega })}^{2}({\langle {\hat{n}}_{{{{{{{{\rm{B}}}}}}}}}\rangle }^{2}+2\langle {\hat{n}}_{{{{{{{{\rm{B}}}}}}}}}\rangle +1)}$ ($\langle {\hat{n}}_{{{{{{{{\rm{B}}}}}}}}}\rangle$ is the average number of excited bosons in the dynamics) and ${\omega }^{* }=\omega -{g}_{q}^{2}/\omega$. Aside from renormalization of the electron and phonon energy scales, we see that the electron density, at ${{{{{{{\mathcal{O}}}}}}}}\{{g}_{q}/\omega \}$, and double occupancy, at ${{{{{{{\mathcal{O}}}}}}}}\{{({g}_{q}/\omega )}^{2}\}$, couple to the squeezed-phonon density. This Hamiltonian is exact to ${{{{{{{\mathcal{O}}}}}}}}\{{g}_{q}/\omega \}$, and approximate to ${{{{{{{\mathcal{O}}}}}}}}\{{({g}_{q}/\omega )}^{2}\}$ (and higher orders). See Supplementary Note 2 for details of the derivation and approximations employed. For the time dependence of electronic operators ${\hat{O}}_{{{{{{{{\rm{e}}}}}}}}}$ measured in the original frame, we derive in a similar approximation (details in Supplementary Note 2) a theory in the squeezed frame in which ${\hat{O}}_{{{{{{{{\rm{e}}}}}}}}}$ transforms as ${\hat{O}}_{{{{{{{{\rm{e}}}}}}}}}\to {e}^{S}{\hat{O}}_{{{{{{{{\rm{e}}}}}}}}}{e}^{-S}$, the initial state as $\left|0\right\rangle \equiv \left|{{{{{{{\mathbf{\Psi }}}}}}}}\right\rangle \to {e}^{S}\left|0\right\rangle$, and ${{{{{{{{\mathcal{U}}}}}}}}}_{{{{{{{{\rm{eff.}}}}}}}}}={e}^{-i{{{{{{{\mathcal{{H}}}}}}}_{{{{{{{{\rm{eff.}}}}}}}}}}}t}$ governs the time evolution. Within this scheme in which terms larger than ${{{{{{{\mathcal{O}}}}}}}}\{{g}_{q}/\omega \}$ are neglected, the equal-time expectation value of ${\hat{O}}_{{{{{{{{\rm{e}}}}}}}}}$ in the squeezed frame becomes

5 $$\langle {\hat{O}}_{{{{{{{{\rm{e}}}}}}}}}(t)\rangle =	\, \left\langle 0\right|{{{{{{{{\mathcal{U}}}}}}}}}_{{{{{{{{\rm{eff.}}}}}}}}}^{{{{\dagger}}} }(t){\hat{O}}_{{{{{{{{\rm{e}}}}}}}}}{{{{{{{{\mathcal{U}}}}}}}}}_{{{{{{{{\rm{eff.}}}}}}}}}(t)\left|0\right\rangle \\ 	 +\left\langle 0\right|{{{{{{{{\mathcal{U}}}}}}}}}_{{{{{{{{\rm{eff.}}}}}}}}}^{{{{\dagger}}} }(t){{{\Gamma }}}_{{\hat{O}}_{{{{{{{{\rm{e}}}}}}}}}}{{{{{{{{\mathcal{U}}}}}}}}}_{{{{{{{{\rm{eff.}}}}}}}}}(t)\left|0\right\rangle ,$$

with ${{{\Gamma }}}_{{\hat{O}}_{{{{{{{{\rm{e}}}}}}}}}}=\left[S,{\hat{O}}_{{{{{{{{\rm{e}}}}}}}}}\right]$. In Fig. 4, we test the predictions of Eq. (5) against the exact results. Not only does the effective theory reproduce the flattening in momentum-resolved *C*_*k*_(*t*) and *S*_*k*_(*t*) (Fig. 4, panels a and b) observed in the exact simulations (Fig. 3), it also provides an overall qualitative proxy for the exact raw and time-averaged quantities 〈*n*_*i*,*↑*_*n*_*i*,*↓*_(*t*)〉, *C*_*π*_(*t*) and *S*_*π*_(*t*) (Fig. 4, panels c–h), even for relatively large *g*_*q*_/*ω* for which the approximations we employ are less justified (we discuss limitations of the effective model in Supplementary Note 2).

**Fig. 4:**
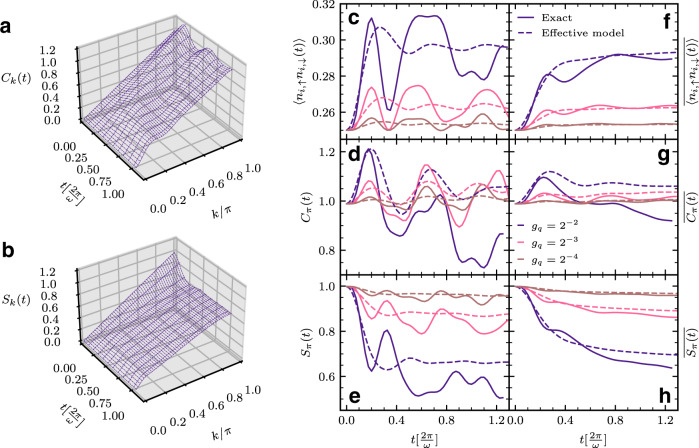
Dynamics of a pumped metal in the effective theory. **a**, **b** Evolution with time of momentum-resolved charge ${C}_{k}(t)={{{{{{{\mathcal{F}}}}}}}}\{{C}_{r}(t)\}$ (**a**) and spin ${S}_{k}(t)={{{{{{{\mathcal{F}}}}}}}}\{{S}_{r}(t)\}$ (**b**) correlation functions (${{{{{{{\mathcal{F}}}}}}}}$ denotes the Fourier transform, ${C}_{r}\equiv \langle {\hat{n}}_{i}{\hat{n}}_{i+r}\rangle -\langle {\hat{n}}_{i}\rangle \langle {\hat{n}}_{i+r}\rangle$ and ${S}_{r}\equiv \langle ({\hat{n}}_{i,\uparrow }-{\hat{n}}_{i,\downarrow })({\hat{n}}_{i+r,\uparrow }-{\hat{n}}_{i+r,\downarrow })\rangle$) for *g*_*q*_ = 0.25 and *ω* = *π*/2 in the effective model given by Eqs. (4) and (5) from iTEBD simulations. **c**–**h** Dependence on time of raw (**c**–**e**) and time-averaged (**f**–**h**) double occupancy $\langle {\hat{n}}_{i,\uparrow }{\hat{n}}_{i,\downarrow }(t)\rangle$, *π*-charge *C*_*π*_(*t*) and *π*-spin *S*_*π*_(*t*) correlations for various *g*_*q*_ at *ω* = *π*/2 in the exact model (solid line) and the effective model is given by Eqs. (4) and (5) (dashed line) from iTEBD simulations. A bar label over an observable symbol denotes time averaging: $\overline{\langle \hat{O}(t)\rangle }=\frac{1}{t}\int\nolimits_{0}^{t}{{{{{{{\rm{d}}}}}}}}\tau \langle \hat{O}(\tau )\rangle$. We observe good agreement between results obtained in the effective model and the exact simulations of the fully coupled model, including the rapid flattening of charge and spin correlations in the course of the dynamics.

The origin of disorder becomes manifest in the effective model. Noting that in Eq. (5) ${{{\Gamma }}}_{{C}_{k}},{{{\Gamma }}}_{{S}_{k}}=0$ because the charge and spin correlations are conserved under the squeezing transformation implies that the exact dynamics is approximately captured by an effective theory that conserves the squeezed-phonon occupations. This effective theory thus encodes dynamics of the electrons within independent trajectories of different squeezed-phonon configurations in an ensemble given by an initial Poisson-distributed linear combination that describes the *t* = 0 state (now in the squeezed basis) and is thus formally equivalent to the disorder-averaged dynamics of an electronic system quenched in a random, static Poisson-distributed potential determined by the initial state occupations. Charge and spin correlations, by construction, possess no coherence between different squeezed-phonon sectors, and thus very quickly flatten in the course of the dynamics. The exact electronic behavior on transient timescales is therefore dominated by a large degree of effective disorder despite that the initial state and the Hamiltonian in both squeezed and unsqueezed frames are disorder free.

Note that, however, while this effective model remains valid on intermediate timescales, higher-order terms in *g*_*q*_/*ω*, neglected in our treatment, will eventually become important, possibly leading at long times to deviations from the above behavior. Nonetheless, our numerics seem to suggest evolution towards a state with a large disorder that remains robust for extended timescales. We provide in Supplementary Note 3 a complementary treatment of electronic disorder at later times in the unsqueezed frame based on the dynamics induced by phonon decoherence. This disordered behavior persists despite the attractive electron density–density interaction term of ${{{{{{{\mathcal{{H}}}}}}}_{{{{{{{{\rm{eff.}}}}}}}}}}}$, which, at least in 1D, implies that the system lies within a regime far from the superfluid transition^40^.

The picture we obtain here indicates that a translationally uniform system excited by a spatially uniform field governed by electron–phonon nonlinearity will flow towards a state characterized by a high level of randomness in absence of quenched disorder. This behavior was noted in ref. ^13^ based on an analysis of phonon decoherence (see also Supplementary Note 3) and has become a subject of major theoretical interest within the field of disorder-free localization^24–27^ (see also refs. ^41–43^). In this regard, our effective theory reveals a mechanism operative in experiments for dynamically induced disorder reminiscent of that found in the context of the particular lattice gauge theory models studied in refs. ^27–31^. These models describe the coupling of fermions to background gauge fields modeled as spin degrees of freedom, in which a duality transformation^44,45^ maps the Hamiltonian onto one with conserved gauge charge configurations and the gauge charge couples directly onsite to the fermion occupation. Time evolution with this manifestly translationally invariant Hamiltonian of an initial product state of fermions and gauge spins, equivalent to a linear superposition over different superselection gauge charge configurations, exhibits disorder-free localization due to self-averaging of observables over the different initial gauge configurations. In contrast to these models, our theory reveals that an approximate effective model governed by similar behavior dominates the exact dynamics of the quenched electron–phonon system on extended timescales. Thus, our work paves a way towards the physical realization of disorder-free localization in current pump-probe experiments. Furthermore, the emergence of an attractive Hubbard interaction in the effective model presents an unexplored avenue within the context of disorder-free localization to study the competition between disorder and attractive interactions in the dynamics of spinful fermionic systems.

### Comparison with a linearly coupled electron–phonon model: dynamically induced strong-coupling behavior from nonlinear electron–phonon coupling

**Fig. 5:**
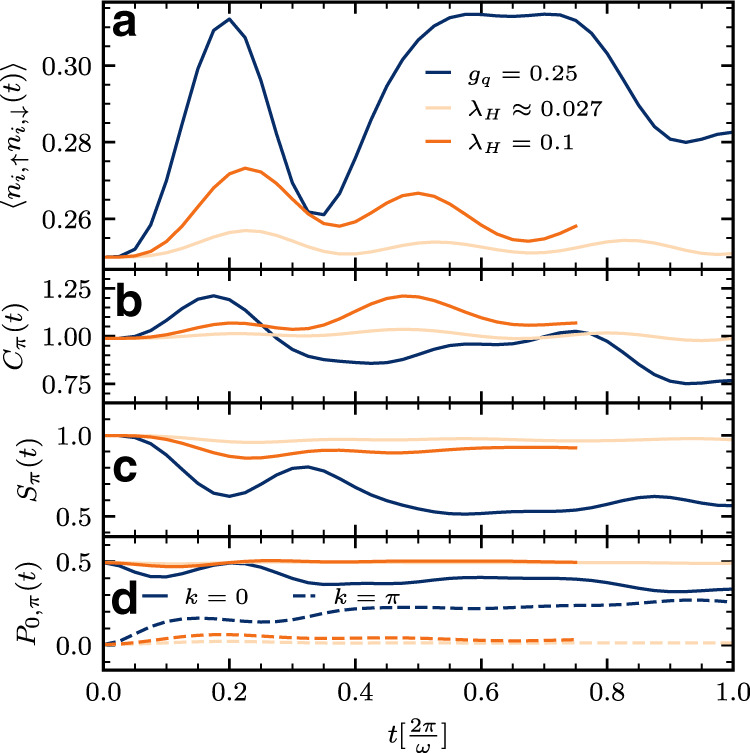
Dynamical response in the quadratically coupled model versus in the Holstein model. A comparison of the pump-induced dynamics in the quadratic-coupling model with coupling constant *g*_*q*_ to that of the Holstein model with coupling constant *g*_H_ and dimensionless effective coupling parameter ${\lambda }_{{{{{\mathrm{H}}}}}}=\frac{{g}_{{{{{\mathrm{H}}}}}}^{2}}{2\omega J}$ for appropriately selected values of the couplings and *ω* = *π*/2 (see text and Supplementary Note 4 for more details) reveals that the driven quadratic model induces a more appreciably enhanced double occupancy (**a**) and causes a greater response in electron correlations (**b**–**d**) including the flattening of pairing tendencies (**d**) than its Holstein model counterpart.

We contrast the dynamics of our nonlinear model to that of the (linear) Holstein model (which cannot be driven by a light pulse in an inversion symmetric system). We use two methods to choose an appropriate coupling strength in the Holstein model corresponding to a given coupling strength of the quadratic model against which we perform a comparison, see Supplementary Note 4 for details. In one approach, we choose the Holstein coupling that yields the same equilibrium ground state double occupancy as in the quadratic model. In the other, the Holstein coupling is chosen to produce the same double occupancy as that obtained analytically from a disentangling transformation that serves as a low-energy description of the dynamics (Eq. (4)). Both methods of comparison show that even a relatively large nonlinear coupling such as *g*_*q*_ = 0.25, proximate to the oscillator instability threshold, gives rise in equilibrium to weak-coupling behavior. In contrast, Fig. 5 shows that the quadratic model exhibits a much stronger dynamical response to the pump, displaying both a large enhancement of double occupancy (panel a) and large-amplitude dynamics in momentum-resolved electronic correlations (panels b–d) including flattening of *P*_*k*_(*t*) (panel d). This is in sharp contrast to the behavior of the Holstein model and implies that dynamics of nonlinear coupled electron–phonon systems operative in pump-probe experiments afford nonequilibrium pathways to correlated physics unavailable in the static limit and which lies outside the frame of conventional theoretical models.

## Discussion

Prior studies of nonlinear electron–phonon dynamics have relied on approximate low-energy treatments. Our exact numerical approach to spatially resolved dynamics of a pumped nonlinear electron–phonon systems fills an urgent need. We use iTEBD to provide a detailed exact analysis of short-time (up to $t \sim \frac{2\pi }{\omega }$) dynamics of an infinite nonlinear electron–phonon coupled metal upon coherent excitation of vibrational modes by light. We supplement this by direct Krylov propagation of small systems to asymptotically long times. We explicitly describe the flow towards a correlated electron–phonon steady state at long times, the indication of which already manifests on short timescales. Remarkably, although we consider a spatially uniform system evolving after application of a spatially uniform pump field, the key feature of the long-time state is the appearance of properties consistent with a high degree of effective disorder that dominates the physical behavior, unveiling an intriguing connection to the scenario of disorder-free localization^27,28^. These properties are a consequence of the quasi-conserved squeezed-phonon constants of motion that effectively govern the time evolution of the initial linear superposition state and the very rapid loss of coherence of the phonons, which we found to be directly tied to the buildup of disorder, implying that the intermediate- and long-time state is an incoherent superposition of different oscillator configurations on different sites. These incoherent phonon configurations result in a dynamic effective disorder potential for the electrons, which leads to the suppression of the (power-law) quasi-long-range charge, spin and pairing correlations. Analysis of the energy redistribution amongst the different system subsectors and of electron and phonon distribution functions of the long-time state obtained in finite-size systems, presented in Supplementary Note 3, suggests that the terminal state obtained in finite-size simulations may not be thermal. Determining the fate of the established long-time entangled electron–phonon state in which the phonons in effect provide strong onsite potential fluctuations that substantially broaden all momentum–space distribution functions and fully disentangling the contributions of electron heating from localization due to the transient phonon-induced disorder to this entangled electron–phonon state are beyond the scope of this paper, and are left to future work. However, the results of this paper establish that the pump-activated transient phonon-induced disorder in electron dynamics presents an opportunity to explore the interplay between correlations and randomness in out-of-equilibrium electronic matter.

A crucial question relates to the possibility of pump-induced superconductivity as predicted in ref. ^13^. In our calculations no evidence for superconductivity is found and we only find weak evidence for charge density wave correlations for very short time delays; the results are more consistent with the system falling within the disorder-dominated Anderson insulating regime of the phase diagram presented in ref. ^13^. One possibility would be that superconducting and density wave regimes either do not exist or are not accessible with the current pump protocol (perhaps because the pump transfers too much energy to the electronic subsystem). A second possibility would be that the 1D model considered here disfavors superconductivity. In fact, it has been shown that quantum fluctuations can destroy superconductivity in dirty superconductors below a mobility threshold^46^. In one dimension, all single-particle states are localized in presence of a static disorder potential. Despite that in 1D systems superconductivity can overcome the localizing tendency of disorder to some extent^47^, the effects of the disorder are stronger than in higher dimensions. The accurate simulation of pump-induced dynamics in higher-dimensional systems in the thermodynamic limit faces challenges, but is urgently needed.

The quadratic model reacts more strongly to a pump than the linear Holstein model, highlighting the importance of this mechanism in pump-probe experiments, e.g., ref. ^48^. Questions such as the consideration of additional electron–vibration interactions consistent with inversion symmetry^20,49^, which may aid in the stabilization of a transient superconducting state, as well as how the electron–phonon steady state exposed in this work manifests experimentally are also important open challenges and call for the development of new tools for the study of out-of-equilibrium nonlinear electron–phonon problems. An intriguing possibility is to use the information obtained here about the properties of the long-time state to motivate a variational ansatz in order to simulate the dynamics.

## Methods

We study pump-induced dynamics via exact numerical simulations of the nonlinear model coupled with an effective theory derived within a treatment formally similar to a linear response theory in a low-order expansion in powers of *g*_*q*_/*ω*.

### Details of exact numerical simulations of the nonlinear electron–phonon system

We simulate the time evolution of $\left|{{{{{{{\mathbf{\Psi }}}}}}}}\right\rangle$ representing the metal on an infinite chain irradiated at initial time *t* = 0 by a pump via the iTEBD algorithm^38^ utilizing the TeNPy Library^50^. We use *d*_*ν*_ = 12 phonon states to represent the local phonon Hilbert space. We allow the bond dimension *χ* to grow without saturation in the iTEBD time evolution, and converge our results with respect to the truncation error *ϵ*_TEBD_. This allows access to time *t* ~ 5*J* for which we find *ϵ*_TEBD_ = 10^−3.5^ achieves satisfactory convergence. We refer the reader to Supplementary Note 5 for more information. To shed light on the long-time behavior, we also propagate the initial state using direct Krylov subspace methods for finite system sizes *L* = 3 − 6 with *d*_*ν*_ = 8, 10, 12 and twisted boundary conditions, see Supplementary Note 5 for more details.

### Details of the effective model obtained within a low-order expansion in *g*_*q*_/*ω*

We derive an effective model within a framework similar to linear response, consistently incorporating contributions of ${{{{{{{\mathcal{O}}}}}}}}\{{g}_{q}/\omega \}$, with judiciously selected ${{{{{{{\mathcal{O}}}}}}}}\{{({g}_{q}/\omega )}^{2}\}$ corrections (e.g., the effective electron density–density interaction term). This theory, strictly valid to ${{{{{{{\mathcal{O}}}}}}}}\{{g}_{q}/\omega \}$, qualitatively captures the exact behavior of the time-evolved initial state in infinite systems obtained using iTEBD. We simulate the dynamics governed by the effective model by time-evolving $\left|0\right\rangle \equiv \left|{{{{{{{\mathbf{\Psi }}}}}}}}\right\rangle$ under the action of ${{{{{{{{\mathcal{U}}}}}}}}}_{{{{{{{{\rm{eff.}}}}}}}}}(t)$ in Eq. (5) using iTEBD, employing *d*_*ν*_ = 12 local squeezed-phonon states and allowing *χ* to grow without saturation in the time evolution, while converging results with respect to *ϵ*_TEBD_. This allows access to time *t* ~ 5*J* for which we find *ϵ*_TEBD_ = 10^−3.5^ achieves satisfactory convergence. Details of the derivation of the effective model and additional discussion of the dynamics are presented in Supplementary Note 2 and Supplementary Note 3.

## Supplementary information

Supplementary Information

## Data Availability

All data are available upon reasonable request.

## Code availability

All the custom codes used in this work are available upon reasonable request.
