# Supplementary material for: Phonon-induced disorder in dynamics of optically pumped metals from nonlinear electron-phonon coupling
Source: Nat Commun. 2021 Oct 4;12:5803. doi: 10.1038/s41467-021-26030-3 (PMC8490472; doi:10.1038/s41467-021-26030-3)
Supplement: Supplementary file 1 — Supplementary Information [file 41467_2021_26030_MOESM1_ESM.pdf]

SUPPLEMENTARY INFORMATION
for
"Phonon-induced disorder in dynamics of optically pumped metals from non-linear electron-phonon coupling"

John Sous,[1,*] Benedikt Kloss,[2] Dante M. Kennes,[3,4] David R. Reichman,[2,†] and Andrew J. Millis[1,5,‡]

[1] *Department of Physics, Columbia University, New York, New York 10027, USA*
[2] *Department of Chemistry, Columbia University, New York, New York 10027, USA*
[3] *Institut für Theorie der Statistischen Physik, RWTH Aachen, 52056 Aachen, Germany and JARA - Fundamentals of Future Information Technology*
[4] *Max Planck Institute for the Structure and Dynamics of Matter and Center for Free-Electron Laser Science, 22761 Hamburg, Germany*
[5] *Center for Computational Quantum Physics, Flatiron Institute, 162 5th Avenue, New York, New York 10010, USA*

(Dated: August 30, 2021)

Supplementary Note 1. STABILITY OF THE QUADRATIC ELECTRON-PHONON MODEL

In this section, we derive the stability condition of the quadratically coupled electron-phonon model we consider. The Hamiltonian of the model reads

$$\mathcal{H} = \mathcal{H}_{\text{e}} + \mathcal{H}_{\text{ph}} + \mathcal{V}_{\text{e-ph}}, \tag{1}$$

where

$$\mathcal{H}_{\text{e}} = -J \sum_{i,\sigma} c^{\dagger}_{i,\sigma} c_{i+1,\sigma} + \text{H.c.}, \tag{2}$$

$$\mathcal{H}_{\text{ph}} = \omega \sum_{i} \left(b^{\dagger}_{i} b_{i} + \frac{1}{2} \right), \tag{3}$$

$$\mathcal{V}_{\text{e-ph}} = g_q \sum_{i} (\hat{n}_i - 1)(b^{\dagger}_{i} + b_i)^2. \tag{4}$$

A stable harmonic oscillator mode localized on a given site implies an oscillator stiffness $K > 0$. To derive the condition for stability of the coupled electron-phonon system, we rewrite $\mathcal{H}$ in terms of the harmonic oscillator displacement $\hat{X}_i$ and momentum $\hat{P}_i$ operators. We make use of the relation ($\hbar = 1$)

$$b_i = \sqrt{\frac{M\omega}{2}} (\hat{X}_i + \mathrm{i}\frac{1}{M\omega}\hat{P}_i), \tag{5}$$

where M is the oscillator mass, to obtain

$$\mathcal{H}_{\text{ph}} = \sum_{i} \frac{1}{2} K \hat{X}^2_i + \sum_{i} \frac{1}{2M} \hat{P}^2_i \tag{6}$$

$$\mathcal{V}_{\text{e-ph}} = 2\frac{g_q}{\omega} K \sum_{i} (\hat{n}_i - 1)\hat{X}^2_i, \tag{7}$$

in which we used $K = \omega^2 M$. Thus, the quadratic electron-phonon coupling renormalizes the oscillator stiffness on any

* js5530@columbia.edu
† drr2103@columbia.edu
‡ Correspondence and requests for materials should be addressed to A. J. M.: ajm2010@columbia.edu

given site

$$K \to K\Big[1 + 4\big(\hat{n} - 1\big)\frac{g_q}{\omega}\Big]. \tag{8}$$

Demanding that $K > 0$, we arrive at the stability condition of the electron-phonon model:

$$\big|g_q\big| < \frac{\omega}{4}. \tag{9}$$

For spinless electrons $\hat{n} - 1 \to \hat{n} - 1/2$ and the stability condition, then, is $\big|g_q\big| < \frac{\omega}{2}$.

Supplementary Note 2. EFFECTIVE MODEL

In this section, we derive in detail the effective model for the disordered dynamics we obtain within a treatment, reminiscent of linear response, based on a low-order expansion in g_q/ω (and valid for arbitrary quench amplitude). We discuss our approximations and limitations of the model.

As discussed in the main text, we move to a rotating squeezed frame, in which we derive a response theory to leading order in g_q/ω. By direct comparison against the exact results we show that the effective model captures the main qualitative features of the exact model dynamics.

A. Squeezing transformation

Kennes et al. [13] found a transformation that rescales the phonon coordinate, rotating the Hamiltonian Supplementary Eqs. (1)-(4) into a frame in which the electrons and phonons are approximately decoupled. The electron density-dependent transformation $\mathcal{H} \to \tilde{\mathcal{H}} = U\mathcal{H}U^\dagger$, with $U = e^S$, $S = -\sum_j \frac{1}{2}\zeta_j(b_j^\dagger b_j^\dagger - b_j b_j)$ and squeezing parameter $\zeta_i = -\frac{1}{4}\ln[1 + 4\frac{g_q}{\omega}(\hat{n}_i - 1)]$, yields

$$\begin{aligned}\beta_i^\dagger &\equiv e^S b_i^\dagger e^{-S} = \cosh(\zeta_i) b_i^\dagger + \sinh(\zeta_i) b_i,\\ \beta_i &\equiv e^S b_i e^{-S} = \cosh(\zeta_i) b_i + \sinh(\zeta_i) b_i^\dagger.\end{aligned} \tag{10}$$

Here $\beta_i^\dagger$ creates a squeezed phonon state on site i. Under this transformation, $\mathcal{H}_{\rm ph} + \mathcal{V}_{\rm e\text{-}ph}$ is recast into a form completely diagonal in the squeezed phonon occupation basis:

$$\tilde{\mathcal{H}} = \tilde{\mathcal{H}}_{\rm e} + \sum_i \omega\sqrt{1 + 4\frac{g_q}{\omega}(\hat{n}_i - 1)}\,(\beta_i^\dagger\beta_i + \frac{1}{2}), \tag{11}$$

where $\tilde{\mathcal{H}}_{\rm e} = e^S\mathcal{H}_{\rm e}e^{-S}$ is the squeezed electronic Hamiltonian (we discuss a treatment of this term below). Our formulation of the problem amounts to a quench in which the non-linear electron-phonon coupling is suddenly switched on. (We have verified that the quench dynamics of electronic correlations obtained from the time evolution of the initial product state of electrons and phonons exactly resembles that obtained from an initial correlated electron-phonon state on the accessible timescales in iTEBD.) In the original frame this generates phonon quanta in the dynamics due to the off-diagonal phonon terms in the coupling (Supplementary Eq. (4)). In contrast, the squeezed Hamiltonian conserves the boson number, which, however, couples to the square-root of the electron density operator. We will see next that we can take advantage of this form in order to understand the nature of the dynamics of electronic correlations.

The ratio of energy scales g_q/ω arises naturally in the squeezed frame. This provides an opportunity to expand the interaction term in the squeezed Hamiltonian directly in orders of g_q/ω. In the limit $g_q \ll \omega$, we Taylor expand to

$\mathcal{O}\{(g_q/\omega)^2\}$ and find

$$\tilde{\mathcal{H}} \approx \tilde{\mathcal{H}}_{\rm e} + \sum_i \big[\omega - 2\big(g_q + \frac{g_q^2}{\omega}\big)\big](\beta_i^\dagger\beta_i + \frac{1}{2}) + 2\big(g_q + \frac{g_q^2}{\omega}\big)\sum_i \hat{n}_i(\beta_i^\dagger\beta_i + \frac{1}{2}) - 4\frac{g_q^2}{\omega}\sum_i \hat{n}_{i,\uparrow}\hat{n}_{i,\downarrow}(\beta_i^\dagger\beta_i + \frac{1}{2}). \tag{12}$$

To this order the electron-phonon coupling is completely local and the squeezed phonon number on each site is conserved. The second term describes a squeezed phonon bath term with a renormalized energy scale $\tilde{\omega} = \big[\omega - 2\big(g_q + \frac{g_q^2}{\omega}\big)\big]$, which includes corrections at both $\mathcal{O}\{g_q/\omega\}$ and $\mathcal{O}\{(g_q/\omega)^2\}$. The third term shows that the phonon occupation on site i changes the electron's local chemical potential, again at both orders. This gives rise to disorder, static at this level of approximation, due to the nature of the initial state (as explained within our effective theory, see below and main text). Higher-order terms neglected in the transformation will lead to the evolution of $\beta_i^\dagger\beta_i$, changing the disorder from static to dynamic. Phonons also mediate an effective local electron-electron attraction (fourth term) that appears first at second order.

We have so far postponed a treatment of the $\tilde{\mathcal{H}}_{\rm e}$. To proceed, we evaluate this term in the electron occupation number basis:

$$\tilde{\mathcal{H}}_{\rm e} \equiv \tilde{\mathcal{H}}_{\rm e}[\{n_i, n_j\}] = -J\sum_{\langle ij\rangle,\sigma} c_{i,\sigma}^\dagger c_{j,\sigma} e^{(\zeta[n_i+1]-\zeta[n_i])\mathcal{B}_i} e^{(\zeta[n_j-1]-\zeta[n_j])\mathcal{B}_j}, \tag{13}$$

where $\langle ij\rangle$ refers to $j = i \pm 1$ and $\mathcal{B}_i \equiv \frac{1}{2}(\beta_i^\dagger\beta_i^\dagger - \beta_i\beta_i)$. Expanding the logarithm in the definition of ζ_i to $\mathcal{O}\{g_q/\omega\}$, we find $\zeta[n_i \pm 1] - \zeta[n_i] = \mp g_q/\omega$. Thus, to $\mathcal{O}\{g_q/\omega\}$, the exponential factors in Supplementary Eq. (13) reduce to unity and we retrieve the original untransformed electronic hopping term. To incorporate corrections in the electronic hopping due to the squeezing transformation we must expand the exponentials to next order:

$$\begin{aligned} e^{(\zeta[n_i+1]-\zeta[n_i])\mathcal{B}_i} e^{(\zeta[n_j-1]-\zeta[n_j])\mathcal{B}_j} \approx 1 &+ (\zeta[n_i+1]-\zeta[n_i])(\zeta[n_j-1]-\zeta[n_j])\mathcal{B}_i\mathcal{B}_j \\ &+ \frac{1}{2}\big((\zeta[n_i+1]-\zeta[n_i])^2\mathcal{B}_i^2 + (\zeta[n_j-1]-\zeta[n_j])\mathcal{B}_j^2)\big). \end{aligned}$$

Invoking an inelastic approximation in which one neglects correlations between phonon states on different sites and thus the $\mathcal{B}_i\mathcal{B}_j$ term (this can be rationalized either by simply noting that the initial phonon state is a product over site wavefunctions whose inter-site correlations ought to be unimportant at very early times or by utilizing an incoherent approximation to the phonon density matrix [13]), and evaluating the last term in the initial coherent phonon state, we find a contribution at $\mathcal{O}\{(g_q/\omega)^2\}$: $-\frac{1}{2}(\frac{g_q}{\omega})^2(\langle\hat{n}_{\rm B}\rangle^2 + 2\langle\hat{n}_{\rm B}\rangle + 1)$, where $\langle\hat{n}_{\rm B}\rangle = |\alpha|^2$ is the expectation value of $\hat{n}_{\rm B} = \beta^\dagger\beta$ in the initial state. Re-summing all similar contributions in the exponential, we find

$$J^* = Je^{-\frac{1}{2}(\frac{g_q}{\omega})^2(\langle\hat{n}_{\rm B}\rangle^2+2\langle\hat{n}_{\rm B}\rangle+1)}.$$

This result is exact to $\mathcal{O}\{g_q/\omega\}$ and approximate to $\mathcal{O}\{(g_q/\omega)^2\}$ and higher orders due to the inelastic approximation.

Collecting the various terms, we arrive at the approximate effective Hamiltonian, Eq. (4) of the main text:

$$\begin{aligned} \mathcal{H}_{\rm eff.} = -J^*\sum_{i,\sigma}(c_{i,\sigma}^\dagger c_{i+1,\sigma} + {\rm H.c.}) + \omega^*\sum_i \left(\beta_i^\dagger\beta_i + \frac{1}{2}\right) \\ + 2g_q\sum_i(\hat{n}_i - 1)\left(\beta_i^\dagger\beta_i + \frac{1}{2}\right) - 4\frac{g_q^2}{\omega}\sum_i(\hat{n}_{i,\uparrow} - 1/2)(\hat{n}_{i,\downarrow} - 1/2)\left(\beta_i^\dagger\beta_i + \frac{1}{2}\right), \end{aligned} \tag{14}$$

with $\omega^* = \tilde{\omega} + 2\big(g_q + \frac{1}{2}\frac{g_q^2}{\omega}\big) = \omega - \frac{g_q^2}{\omega}$. Here, we have recast the Hamiltonian in terms of particle-hole symmetric electronic operators. In this form, we see that the density-density interaction and the renormalized phonon terms are suppressed by a factor of $\omega/g_q \gg 1$ relative to the particle-hole symmetric electronic density term responsible for disorder (third term), suggesting that in this limit the density term dominates the dynamics governed by $\mathcal{H}_{\rm eff.}$.

B. Dynamics of observables in the squeezed frame

The form of $\mathcal{H}_{\text{eff.}}$ in Supplementary Eq.(14) (Eq. (4) of the main text) provides a simple view within which to understand the influence of the non-linear electron-phonon coupling on electronic properties. However, in order to expose the intricate details of the dynamics on short and intermediate timescales, one must consider the action of the transformation on the initial state and observables of interest. Consider an observable $\hat{O}$ measured in the original frame $\langle\hat{O}(t)\rangle = \langle 0|\mathcal{U}^\dagger(t)\hat{O}\mathcal{U}(t)|0\rangle$, where $|0\rangle \equiv |\mathbf{\Psi}\rangle$ is the initial state and $\mathcal{U}(t) = e^{-i\mathcal{H}t}$ is the time evolution operator for $\mathcal{H}$, Supplementary Eqs. (1)-(4) (Eq. (1) of the main text). We can obtain $\langle\hat{O}(t)\rangle$ equivalently in the squeezed frame defined by the transformation $U = e^S$ discussed above as $\langle\hat{O}(t)\rangle = \langle\tilde{0}|\tilde{\mathcal{U}}^\dagger(t)\tilde{\hat{O}}\tilde{\mathcal{U}}(t)|\tilde{0}\rangle$, where $|\tilde{0}\rangle = e^S|0\rangle$, $\tilde{\mathcal{U}}(t) = e^{-i\tilde{\mathcal{H}}t}$ and $\tilde{\hat{O}} = e^S\hat{O}e^{-S}$ are now in the rotated frame. Evaluating $\langle\hat{O}(t)\rangle$ in this fashion comes with advantages. The squeezed Hamiltonian conserves the squeezed phonon number, affording an analysis of the dynamics in terms of these constants of motion. In fact, we can make use of the approximations derived above and systematically consider the dynamics under the action of $\mathcal{H}_{\text{eff.}}$ instead of $\tilde{\mathcal{H}}$ in the limit $g_q \ll \omega$ by making use of the following. We first write $\langle\hat{O}(t)\rangle = \langle\tilde{0}|\tilde{\mathcal{U}}^\dagger(t)\tilde{\hat{O}}\tilde{\mathcal{U}}(t)|\tilde{0}\rangle = \langle\tilde{0}|\,\mathrm{G}^\dagger(t)\mathcal{U}_{\text{eff.}}{}^\dagger(t)\tilde{\hat{O}}\mathcal{U}_{\text{eff.}}(t)\mathrm{G}(t)\,|\tilde{0}\rangle$. Here $\mathcal{U}_{\text{eff.}} = e^{-i\mathcal{H}_{\text{eff.}}t}$ and $\mathrm{G}(t) = \mathcal{U}^\dagger_{\text{eff.}}(t)\tilde{\mathcal{U}}(t)$, for which we can derive an integral equation of motion: $\mathrm{G}(t) = 1 - i\int_0^t dt' e^{i\mathcal{H}_{\text{eff.}}t'}(\tilde{\mathcal{H}} - \mathcal{H}_{\text{eff.}})e^{-i\mathcal{H}_{\text{eff.}}t'}\mathrm{G}(t')$. We expand this expression for $\mathrm{G}(t)$ in linear response, retaining terms of $\mathcal{O}\{g_q/\omega\}$. As discussed in the previous subsection, $\mathcal{H}_{\text{eff.}}$ is exact to $\mathcal{O}\{g_q/\omega\}$. Thus, the leading-order terms in $\tilde{\mathcal{H}} - \mathcal{H}_{\text{eff.}}$ are already $\mathcal{O}\{(g_q/\omega)^2\}$. We see that

$$\langle\hat{O}(t)\rangle = \langle\tilde{0}|\mathcal{U}^\dagger_{\text{eff.}}(t)\tilde{\hat{O}}\mathcal{U}_{\text{eff.}}(t)|\tilde{0}\rangle$$

to $\mathcal{O}\{g_q/\omega\}$, and we simply need to consider the transformed initial state and observable under the action of $\mathcal{H}_{\text{eff.}}$ instead of $\tilde{\mathcal{H}}$. Next, note that the transformation itself is parametrized by the ratio g_q/ω in ζ_i, which allows us to simplify this expression by expanding $e^S \approx 1 + S$. Thus, the equal-time expectation value of $\hat{O}$ to $\mathcal{O}\{g_q/\omega\}$ in the squeezed frame is

$$\langle\hat{O}(t)\rangle = \langle 0|\mathcal{U}^\dagger_{\text{eff.}}(t)\hat{O}\mathcal{U}_{\text{eff.}}(t)|0\rangle + \langle 0|\mathcal{U}^\dagger_{\text{eff.}}(t)\Gamma_{\hat{O}}\mathcal{U}_{\text{eff.}}(t)|0\rangle + \langle 0|\mathcal{U}^\dagger_{\text{eff.}}(t)\hat{O}\mathcal{U}_{\text{eff.}}(t)|g\rangle + \langle g|\mathcal{U}^\dagger_{\text{eff.}}(t)\hat{O}\mathcal{U}_{\text{eff.}}(t)|0\rangle\,, \quad (15)$$

where $\Gamma_{\hat{O}} = \left[S, \hat{O}\right]$, and $|g\rangle = S|0\rangle$. This result obtained within a linear response-like treatment consistently incorporates $\mathcal{O}\{g_q/\omega\}$ corrections in the time evolution.

We are interested in the time evolution of the expectation values of electronic operators $\hat{O} = \hat{O}_{\text{e}}$ that depend on the charge or spin density. Noting that charge and spin correlations are conserved under the squeezing transformation ($\Gamma_C, \Gamma_S = 0$) and that terms in the above expression connecting $|0\rangle$ and $|g\rangle$ for $\hat{O} = \hat{O}_{\text{e}}$ vanish at $\mathcal{O}\{g_q/\omega\}$ for real α used throughout this work, we arrive at (see Eq. (5) of the main text)

$$\langle\hat{O}(t)\rangle = \langle 0|\mathcal{U}^\dagger_{\text{eff.}}(t)\hat{O}\mathcal{U}_{\text{eff.}}(t)|0\rangle\,.$$

To $\mathcal{O}\{g_q/\omega\}$, the dynamics of charge and spin correlations can be understood within an effective model in which we simply time evolve the initial phonon coherent state (now in the squeezed basis) under the action of $\mathcal{H}_{\text{eff.}}$.

As we show in the main text, this effective model captures in a qualitative and sometimes semi-quantitative manner the behavior found in the exact results obtained in the unrotated frame. This simple model, however, provides evidence that the exact dynamics of the initial phonon coherent state is dominated by physical behavior given by its time evolution with $\mathcal{H}_{\text{eff.}}$, which results in an ensemble of trajectories of independent conserved squeezed phonon configurations, and because the initial state is a Poisson linear superposition over phonon number states, this can be viewed as exactly equivalent to the disorder-averaged dynamics of a random system quenched to Poisson-distributed disorder, as has been established for models with binary disorder [27, 28].

The utility of the effective model as a descriptor of the behavior of electronic correlations may in fact extend to long times, but we have no means of confirming this since we only have access to correlation functions in infinite systems

for which simulations are limited to short times. Below we will discuss a complementary approach within which to understand the dynamics of electronic correlations in the original untransformed frame, which lends support to the persistence of disorder to longer times.

Supplementary Note 3. SUPPLEMENTARY DISCUSSION OF DYNAMICS: DECOHERENCE AND HEATING

In this section, we discuss supplementary details of the dynamics pertaining to decoherence and its influence on electronic dynamics, and heating.

A. Dephasing phonon-induced dynamics

Similar to the approach of Ref. [13], we attempt to understand the influence of phonon coherence on the electronic dynamics. The main result we find is that the onsite phonon reduced density matrix in the phonon-number basis rapidly relaxes from its initial coherent state to a predominantly diagonal matrix, the elements of which form a unimodal distribution very similar to the Poisson distribution that describes the eigenvalues of the initial phonon density matrix. This persistence of the diagonal character of the initial state phonon reduced density matrix accompanied by its rapid dephasing means that the approximation of the phonon distribution as an incoherent average over Poisson-distributed occupation-number eigenstates is reasonable, and lends support to the idea that disordered electron dynamics is intimately related to the nature of the initial state being a Poisson-distributed linear combination over phonon-number states.

To access these effects in the dynamics in the original unsqueezed frame from the exact data, we devise an approximate semi-classical *in silico* approach, which, using the exact phonon reduced density matrix extracted from the simulations, reproduces qualitatively the flattening in charge correlations. This *in silico* approach in which one extracts information from the exact behavior of the phonons in order to reproduce the qualitative features of the dynamics of electronic correlations is not exact, but serves as a perspective on the influence of phonon decoherence on the electrons, complementary to the results obtained within the effective model presented in the main text.

We analyze the loss of coherence with time of the onsite oscillator reduced density matrix $\rho^R_{\mathrm{ph}}(t)$ in the phonon occupation-number basis. We study the quantity $\eta(t) \equiv \sum_{\nu\neq\mu} |\rho^R_{\mathrm{ph}_{\nu,\mu}}(t)| / \sum_{\nu\neq\mu} |\rho^R_{\mathrm{ph}_{\nu,\mu}}(0)|$ (ν and μ are states of different phonon occupation number) as a measure of decoherence (Supplementary Fig. 1, panels a and c). We find that $\eta(t)$ drops sharply from its initial value of unity corresponding to the pure initial phonon state to below 50% at $t \sim 4J^{-1}$ and to vanishingly small values in the long-time limit. This implies that $\rho^R_{\mathrm{ph}}(t)$ evolves from its initial pure coherent state $|\alpha\rangle\langle\alpha|$ to a mixed state that is predominantly diagonal in the phonon-number basis, signalling rapid dephasing of states with different phonon occupation number. The dephased configuration exhibits a unimodal distribution of diagonal matrix elements, which closely resembles the initial state Poisson distribution. Our numerics reveals a strong sensitivity of the electron dynamics to the approach of ρ^R_{ph} to diagonality, as also corroborated in finite-size systems in which we find the phonon coherence and electronic observables (e.g. energy density) both relax and approach the steady state on the same characteristic timescale $t \sim 5J$ (not shown). This suggests that the diagonal matrix elements of the $\hat{X}^2$ operator can be thought of as a slowly evolving classical dynamical onsite potential for the electrons (see also Fig. 2 of the main text, showing slow evolution with time of $\hat{X}^2$ and its correlation with charge at $t \sim 5J$). We may thus invoke a semi-classical approximation in which we neglect the rapidly decaying and small off-diagonal components of the $\hat{X}^2$ operator $\propto {b^\dagger}^2$ and b^2, and model it as a classical diagonal variable that couples to the electron density in order to understand the influence of the non-linear coupling on the dynamics of electrons in terms of a dephasing phonon-generated disorder, which ultimately destroys the initial state quasi-long-ranged electronic density wave correlations.

To simulate this picture we consider an Anderson model for the dynamics of an initial state of a translation-invariant

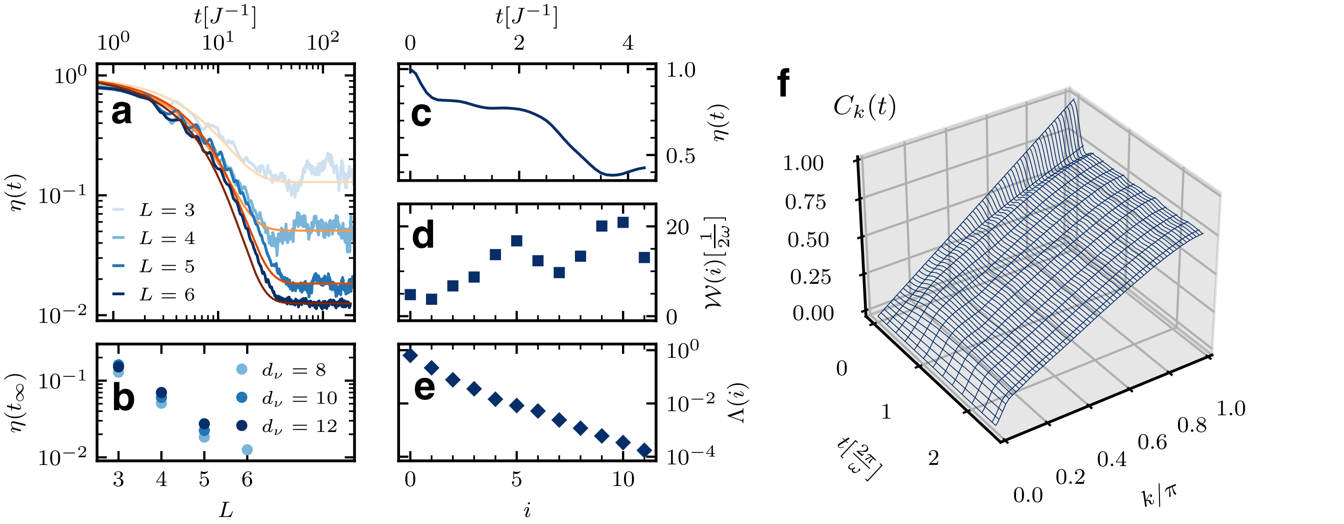

Supplementary Fig. 1. **Dynamics of a metal subjected to a quadratic coupling, dephasing phonon-generated disorder. a** and **b**: Rapid loss of coherence in the onsite phonon reduced density matrix ρ^R_{ph} shown via analysis of $\eta(t) \equiv \sum_{\nu\neq\mu} |\rho^R_{\mathrm{ph}_{\nu,\mu}}(t)| / \sum_{\nu\neq\mu} |\rho^R_{\mathrm{ph}_{\nu,\mu}}(0)|$. This is verified in Krylov propagation of systems of increasing size (**a** and **b**), and can be already observed on short timescales for infinite systems studied by iTEBD (**c**). Thin lines in orange hues are fits of $\eta(t)$ to an exponential decay to a plateau (**a**). In the long-time limit, $\eta(t_\infty)$ approaches increasingly vanishing values with larger system sizes (**b**). **c**, **d** and **e**: We use the approach to diagonality of ρ^R_{ph} in iTEBD simulations (**c**) to invoke a semi-classical approximation in which we treat the phonons classically, as characterized by their reduced density matrix. We extract a disorder potential from the coupled model for an exemplary time $t_q = \frac{2\pi}{\omega}$ via singular value decomposition of ρ^R_{ph}, which we use to evaluate an effective classical disorder potential $\mathcal{W}(i)$ given by the expectation value of $\hat{X}^2$ in the singular vectors $i_{\mathcal{S}(\rho^R_{\mathrm{ph}})}$ (**d**), and weighed by the probability distribution $\Lambda(i)$ given by the singular values (**e**). **f**: We model the dynamics of the electrons quenched to the dephasing phonon potential given by $\mathcal{W}(i)$ weighted by the probability distribution $\Lambda(i)$, as prescribed by Supplementary Eq. (16). A free metal subjected to this disorder potential at initial time exhibits, after disorder averaging, a flattening charge correlator $C_k(t)$ with a suppressed peak, qualitatively supporting the result of the fully coupled model observed in Fig. 3 of the main text. We use $g_q = 0.25$ and $\omega = \pi/2$ in the simulations of the fully coupled model used in this figure.

free-electron half-filled metal $|\mathrm{F}S\rangle$ with $k_{\mathrm{F}} = \pi/2$ evolved via a Hamiltonian that includes a static quenched onsite disorder potential extracted from the dephased phonon $\hat{X}^2$ obtained in exact simulations of the fully coupled model at intermediate times:

$$\mathcal{H}_{\mathrm{Anderson}} = -J \sum_{i,\sigma} (c^\dagger_{i,\sigma} c_{i+1,\sigma} + \mathrm{H.c.}) + \sum_i \mathcal{E}_i \hat{n}_i \tag{16}$$

with $\mathcal{E}_i$ drawn from a classical disorder potential $\mathcal{W}(i)$ given by the expectation value of $\hat{X}^2$ in the the singular vectors $i \equiv i_{\mathcal{S}(\rho^R_{\mathrm{ph}})}$ of the phonon reduced density matrix ρ^R_{ph} (i.e., $\mathcal{E}_i \in \mathcal{W}(i) = \left\langle i_{\mathcal{S}(\rho^R_{\mathrm{ph}})} \middle| \hat{X}^2 \middle| i_{\mathcal{S}(\rho^R_{\mathrm{ph}})} \right\rangle$) with weights specified by the probability distribution $\Lambda(i)$ of singular values of ρ^R_{ph} over singular vectors i, see Supplementary Fig. 1, panels c, d and e. We find that the momentum-resolved charge dynamics exhibits a rapid flattening (Supplementary Fig. 1, panel f), bolstering the dephasing phonon-induced disorder picture of electron dynamics in the pumped metal.

B. Estimates of electron heating and phonon relaxation

An issue that arises naturally in the context of pump-probe experiments pertains to electronic heating due to phonon relaxation in the dynamics. Here the initial pump creates an excited phonon state that couples to the electrons, and eventually relaxes by exchanging energy with the electronic and electron-phonon subsectors. Transfer of energy to the electrons may ultimately destabilize transient phases that could have emerged outside of equilibrium. This proceeds in

one of the following ways. Either the system, while still far from global thermal equilibrium, evolves to a different out-of-equilibrium state in which the electronic subsystem heats up to an effective temperature larger than the coherence temperature of the emergent phase, or the system eventually reaches true (global) thermal equilibrium at which point out-of-equilibrium behavior ceases to exist and the system becomes fully characterized by thermal distribution functions. In what follows we provide an analysis, based on numerics of finite size systems, of the asymptotic long-time behavior of the energy redistribution amongst the system subsectors as a function of pump fluence and phonon adiabaticity, and of the asymptotic expectation values of local electronic and phononic observables compared against their thermal expectation values which we use as proxy for the physical temperature of the electronic and phononic subsystems.

1. *Energetics as a function of pump fluence and phonon adiabaticity*

To understand the tendency for electronic heating and phononic relaxation as a function of the pump excitation strength α and the adiabaticity regime set by ω, we study the asymptotic net change in energy density of the electronic, phononic and electron-phonon subsectors in the long-time state obtained in finite-size simulations in Supplementary Fig. 2. The results of Supplementary Fig. 2 can be summarizes as follows.

- Trend of energetics with increasing α:
  Electron heating increases with α. Relaxation of the electron-phonon interaction energy increases with α, and is non-vanishing even at smaller α (this is expected, since the interaction should have a stabilizing contribution). Phonon relaxation vanishes at the smallest α studied. For even smaller α, phonon heating becomes possible since the initial phonon state approaches the phonon vacuum state as $\alpha \to 0$.

- Trend of energetics with increasing ω:
  Electronic heating and phonon relaxation exhibit non-monotonic behavior with ω with large changes in the interval $\omega \in [\pi/\sqrt{2}, \pi]J$. Dependence of these quantities on the system size decreases for larger ω. In this limit, electron heating and phonon relaxation decrease with ω (the latter becomes basically negligible at the largest ω), and the

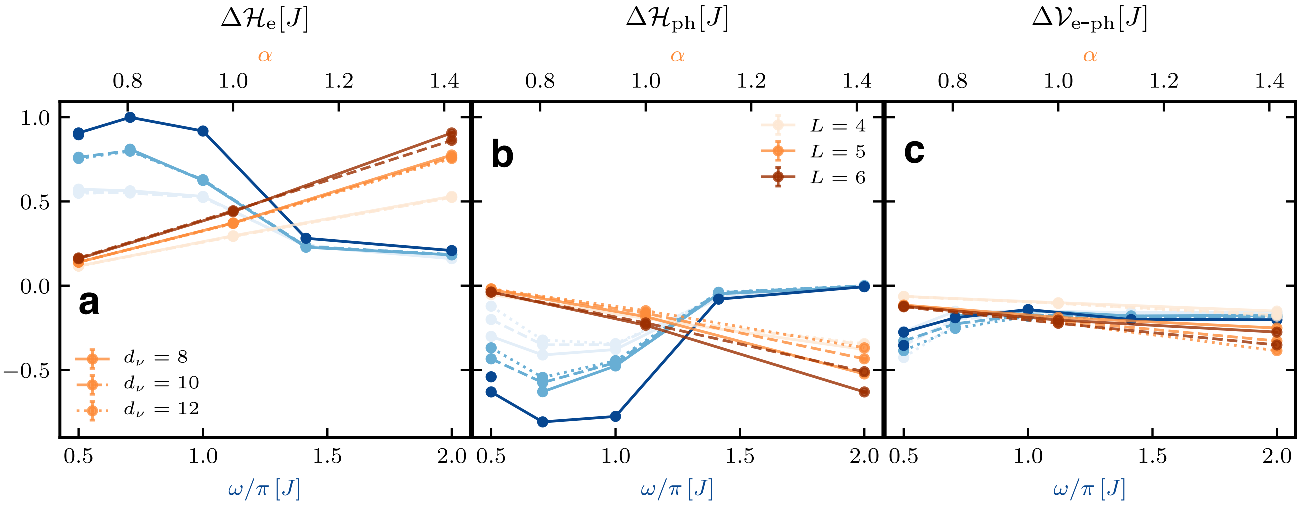

Supplementary Fig. 2. **Net asymptotic change in electronic (a), phononic (b) and electron-phonon (c) energy densities as a function of α, which sets the pump fluence (top horizontal axis), and ω, the phonon frequency (bottom horizontal axis).** Results are obtained from exact Krylov propagation of small systems $L = 4 - 6$ with phonon local Hilbert space dimensions $d_\nu = 8, 10, 12$ ($L = 6$ is restricted to $d_\nu = 8$) to asymptotically long times for the largest electron-phonon coupling $g_q = 0.25$.

electron-phonon interaction energy stabilizes (plateaus) at large ω. The change in the interaction energy is smallest for intermediate values of ω in contrast to the behaviour of the electron and phonon energies.

These results establish that in the adiabatic limit (small ω) and for modest pump fluence ($\alpha = \sqrt{2}$), the long-time state exhibits non-vanishing electron-phonon correlations, accompanied by a net increase in the electronic energy.

2. Long-time state electronic and phononic distribution functions

Given that observables considered in finite-size simulations reach long-time plateaus with fluctuations that are consistent with behavior suggestive of equilibration, it is pertinent to ask whether the long-time steady state is thermal, i.e., if observables have an expectation value consistent with their thermal expectation value at a temperature T that corresponds to the energy density of the initial state. (Note that absence of thermalization in the small system sizes accessible in exact diagonalization (and propagation) is not necessarily guaranteed to hold in the thermodynamic limit.) Here, we use momentum-resolved electronic occupations $\langle \hat{n}_k \rangle$ and onsite phonon populations in the occupation number basis $\langle \hat{n}_\nu \rangle$ of the long-time state to judge whether it can be approximately considered to resemble a thermal state. We contrast these against thermal expectation values which we obtain by computing the full Hamiltonian spectrum using exact diagonalization (ED) of finite-size systems.

In Supplementary Fig. 3 we study $\langle \hat{n}_k \rangle$ in the long-time state. The exact electronic distribution function, at least at large α, resembles a Fermi-Dirac distribution (Supplementary Fig. 3, panel a), however at a significantly lower T than the physical temperature (Supplementary Fig. 3, panel b). We also find that the long-time steady steady state for all α exhibits a more strongly peaked distribution relative to its thermal counterpart obtained at T that corresponds to the initial state energy density (Supplementary Fig. 3, panel b). Note that we could not find good fits to Fermi-Dirac distributions for long-time states obtained for small α values. These observations suggest that the electronic subsystem of the long-time state deviates from a thermal distribution.

In Supplementary Fig. 4 we compute $\langle \hat{n}_\nu \rangle$ in the long-time state. The Poisson-like long-time phonon state found

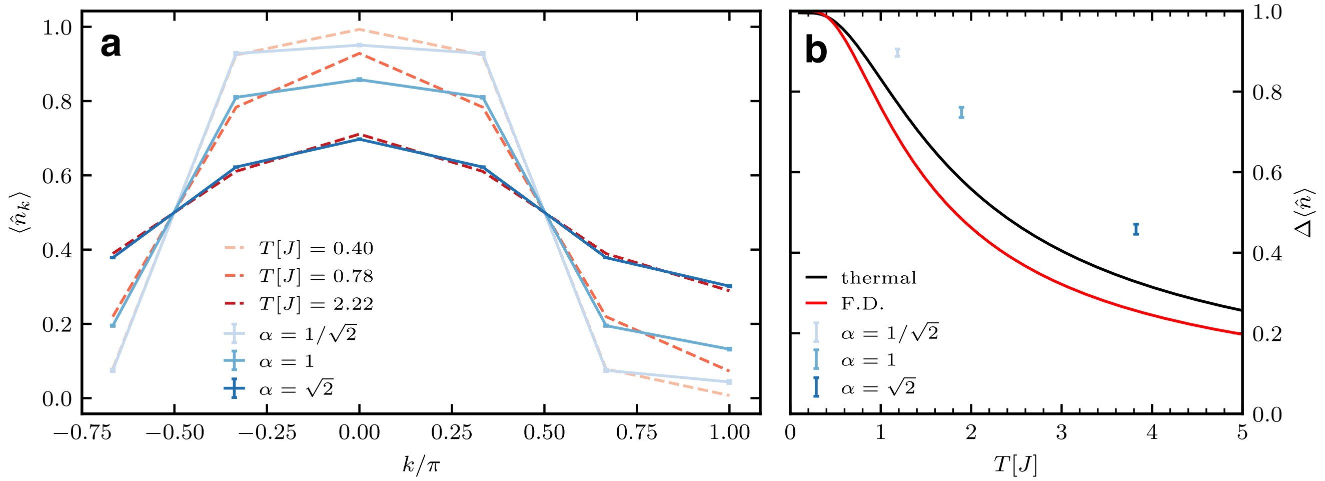

Supplementary Fig. 3. **Electron distribution functions for different initial pump strength α. a**: $\langle \hat{n}(k) \rangle$ of the long-time state from exact Krylov propagation of systems with $L = 6$ and $d_\nu = 8$ (solid lines) and their fits to Fermi-Dirac distributions with temperature T determined from the fit (dashed lines). **b**: $\Delta\langle \hat{n} \rangle \equiv \langle \hat{n}(k=0) \rangle - \langle \hat{n}(k=\pi) \rangle$ for an $L = 4$ ($d_\nu = 8$) system (this fully characterizes $\langle \hat{n}(k) \rangle$ on an $L = 4$ system due to symmetry/conservation laws) in the long-time state obtained from exact Krylov propagation with T determined according to the initial state energy density (blue line error-bar markers; error-bars shows standard deviation of temporal fluctuations). We contrast this against the thermal expectation value of $\Delta\langle \hat{n} \rangle$ at a given T obtained from ED (solid black line) and against the value of $\Delta\langle \hat{n} \rangle$ determined from a Fermi-Dirac distribution at a given T (solid red line) of an $L = 4$ system. All results are for $g_q = 0.25$ and $\omega = \pi/2$.

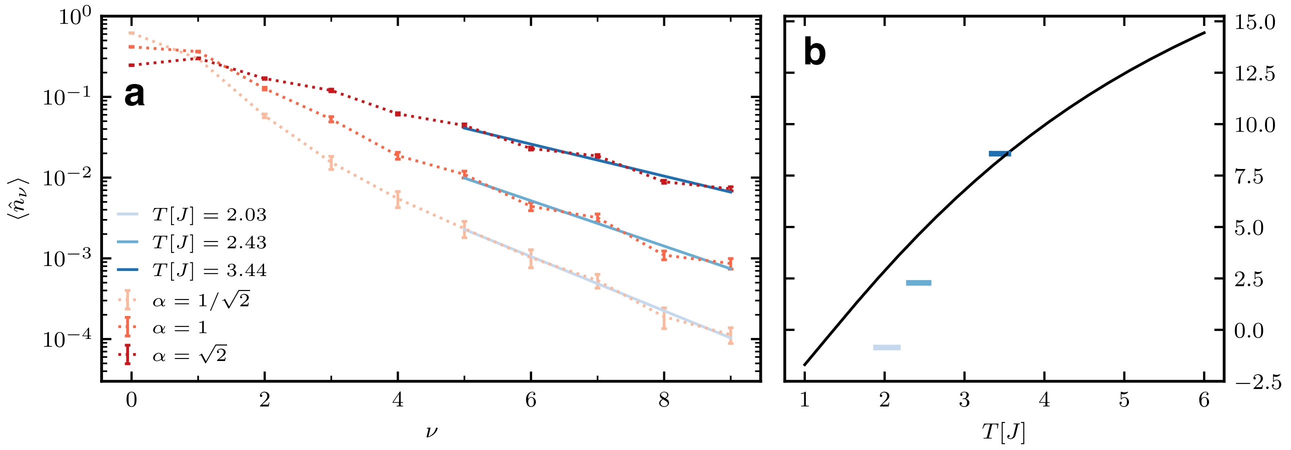

Supplementary Fig. 4. **Phonon distribution functions for different initial pump strength** α**. a**: $\langle \hat{n}_\nu \rangle$ of the long-time state from exact Krylov propagation of systems with $L = 4$ and $d_\nu = 10$ (dotted lines) and the fits of their exponentially decaying tails to Maxwell-Boltzmann distributions with temperature T determined from the fit (solid lines). **b**: Energy density of the initial state for a given α versus T obtained from the fit of the tail of $\langle \hat{n}_\nu \rangle$ for the same α (blue horizontal lines whose length indicates temporal fluctuations obtained as standard deviation of the tail fits over different times in the long-time limit). This is compared to the thermal E as a function of T (solid black line) obtained from the full spectrum of the Hamiltonian computed in ED for $L = 4$, $d_\nu = 10$. All results are for $g_q = 0.25$ and $\omega = \pi/2$.

in finite systems exhibits a maximum in occupation numbers ν and therefore does not fit a thermal distribution. Of course, in a strongly coupled electron-phonon state a thermal phonon distribution is not expected. However, the high-energy tail should still decay in a manner controlled by the equilibrium temperature if the system has approached local equilibrium, and a fit of the exponentially decaying phonon occupation tail to a Maxwell-Boltzmann distribution yields an effective temperature. Carrying out this analysis for the long-time state obtained from exact Krylov propagation, we find that the temperatures extracted from the phonon tail (Supplementary Fig. 4, panel a) overestimates the physical temperature (Supplementary Fig. 4, panel b), except at the largest α. This analysis suggests that the long-time state of the system does not resemble a thermal state. Note that due to the underlying assumption regarding the phonon tail this constitutes less direct evidence of lack of equilibration than the comparison of the electronic distributions to thermal ones discussed above.

The evidence presented above indicates that, at least within the limited system sizes available to exact diagonalization and propagation, the system approaches a non-thermal long-time steady state. Drawing firm conclusions about thermalization from such small system sizes without proper finite-size scaling analysis (the latter being inaccessible to exact numerics) is of course not possible. A more thorough analysis of the existence or absence of thermalization and the associated timescales is left to future work.

Supplementary Note 4. COMPARISON WITH THE LINEARLY COUPLED HOLSTEIN MODEL

In this section we detail the methods we use to decide an appropriate value of the Holstein coupling to compare to a given value of the quadratic coupling.

The Holstein model with electron-phonon coupling $g_H(\hat{n}_i - 1)(b_i^\dagger + b_i)$ can be characterized via the dimensionless coupling $\lambda_H = \frac{g_H^2}{2\omega J}$, the ratio of the ground-state energy in the atomic limit $J = 0$ to that in the free electron limit $g_H = 0$. To compare the Holstein and quadratic models one must find the λ_H most comparable to a given quadratic coupling g_q. We consider the two following approaches to estimate measures of equivalence of coupling strengths:

a. Coupling strengths that give the same double occupancy in the static equilibrium limit:
We find for $\omega = \pi/2$, $g_q = 0.25$ and $g_H = 0.29$ ($\lambda_H \approx 0.027$) yield the same double occupancy in the ground state of

a half-filled chain.

b. Coupling strengths that give the same effective electron-electron interaction obtained from a disentangling transformation:
The Lang-Firsov transformation [51] demonstrates that Holstein phonons mediate an effective electron-electron attraction $U_H = -2\frac{g_H^2}{\omega} \equiv -4\lambda_H J$. The squeezing transformation derived above demonstrates that quadratic phonons mediate an effective electron-electron attraction $U_q = -4\frac{g_q^2}{\omega}\Big(\hat{n}_{\mathrm{B}_i} + 1/2\Big)$ (recall $\hat{n}_{\mathrm{B}_i} = \beta_i^\dagger \beta_i$), see Supplementary Eq. (12). The two models yield the same U when $U_H = U_q$, leading to the condition:

$$\lambda_H = \frac{g_q^2}{\omega J}\Big(\langle \hat{n}_{\mathrm{B}} \rangle + 1/2\Big), \tag{17}$$

where we replaced the phonon number operator by its average over the phonon distribution $\langle \hat{n}_{\mathrm{B}} \rangle$. Since the radiation field creates a coherent state with amplitude α, we take an estimate of $\langle \hat{n}_{\mathrm{B}} \rangle = \alpha^2$ the mean boson number to find λ_H to be used to compare against a given g_q. We thus judge for $\alpha = \sqrt{2}$ and $\omega = \pi/2$ $\lambda_H \approx 0.1$ to be equivalent to $g_q = 0.25$ in the sense that it leads to an effective electron-electron interaction approximately equal to that obtained from the pumped quadratic model (as analyzed within the squeezing transformation).

To summarize, we employ two methods to estimate a value of λ_H to compare to a given value of g_q. One approach assumes the two models are comparable when they yield the same double occupancy in the static ground-state limit, the other compares the undriven Holstein model to the driven quadratic model, making use of analytical results. We can conceptually use these two values of λ_H as approximate lower and upper bounds for comparison against a given value of g_q.

Supplementary Note 5. DETAILS OF NUMERICAL METHODS

In this section we detail the numerical methods and employed convergence parameters used in the simulations of the non-linear electron-phonon model and of the effective model.

A. Details of simulations of the non-linear electron-phonon model

We simulate the time evolution of the initial state $|0\rangle \equiv |\mathbf{\Psi}\rangle$ under the action of the Hamiltonian of the non-linear electron-phonon model Supplementary Eqs. (1)-(4) (Eq. (1) of the main text) to intermediate timescales in infinite systems using iTEBD, and to long timescales in small systems using direct Krylov subspace methods.

1. Details of iTEBD simulations.

The quadratic electron-phonon model connects a phonon state of occupancy ν only to states with $\nu\prime = \nu \pm 2$. These processes conserve phonon parity. We take advantage of this symmetry and parallelize most simulations over even and odd phonon parity subsectors employing up to $d_\nu = 12$ states, see discussion below. We use a fourth-order trotterization scheme for the iTEBD time evolution with time-steps $\mathrm{d}t$. After each time-step, we truncate the Schmidt values of a two-site unit cell state embedded in an infinite system; the discarded Schmidt values squared ϵ_{TEBD} denotes the error due to truncation. We ensure that the bond dimension χ of the time-evolved state after each time-step does not saturate an upper bound we set, which we take to be, for the data points we study, in the range of 3000 – 5000. We converge our results with respect to both $\mathrm{d}t$ and ϵ_{TEBD}, as we explain below.

a. Convergence with respect to dt *and* ϵ_{TEBD}*.* Errors due to dt compete with those due to ϵ_{TEBD}. A sufficiently small dt ensures negligible Trotter error. At the same time, however, it results in more frequent incidents of truncation of the Schmidt values, each of an amount $\sqrt{\epsilon_{\mathrm{TEBD}}}$, thus leading to overall greater Schmidt truncation in order to access a specific desired final time t_f. A sufficiently small ϵ_{TEBD} would eliminate Schmidt errors to within a desirable accuracy, but instead leads to faster growth of entanglement, which scales exponentially in time, and this limits the accessible t_f. To ensure accurate results one needs to converge results with respect to the competing effects due to dt and ϵ_{TEBD}, finding an optimal compromise of a sufficiently small (but not too small) dt to eliminate Trotter error given a reasonably small ϵ_{TEBD} to ensure minimal error due to Schmidt truncation. In Supplementary Fig. 5, we demonstrate convergence for two quantities $P_k(t)$ and $C_k(t)$. The same choices of dt and ϵ_{TEBD} allows convergence of all other quantities considered in this work to the same standard or better. This allows us to approach $t_f \sim 5J^{-1}$.

b. Convergence with respect to d_ν*.* We converge results for electronic and phononic observables with respect to the phonon Hilbert space dimension d_ν within a reasonable accuracy of a few percent. Supplementary Fig. 6 shows satisfactory convergence of representative quantities for $d_\nu = 12$, which we use to obtain the data presented in the main text.

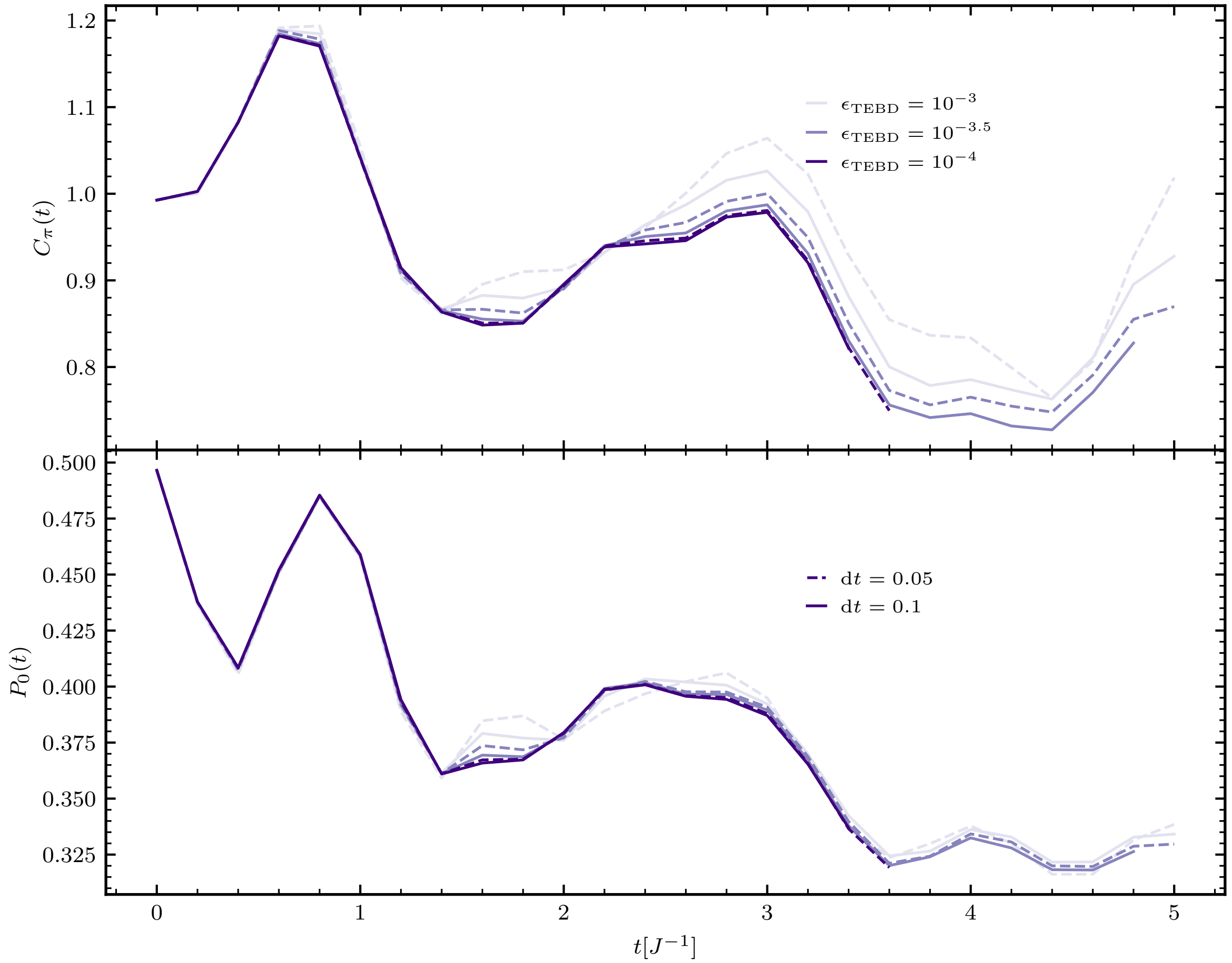

Supplementary Fig.5. **Convergence of time-evolved charge $C_k(t)$ and pairing $P_k(t)$ correlations with respect to truncation error ϵ_{TEBD} and time-step dt used in iTEBD simulations.** We use $g_q = 0.25$ and $\omega = \pi/2$ here, which enables the assessment of convergence for the strongest coupling and smallest phonon frequency considered. We observe satisfactory convergence for $\epsilon_{\mathrm{TEBD}} = 10^{-3.5}$ and d$t = 0.1$ on the accessible timescales.

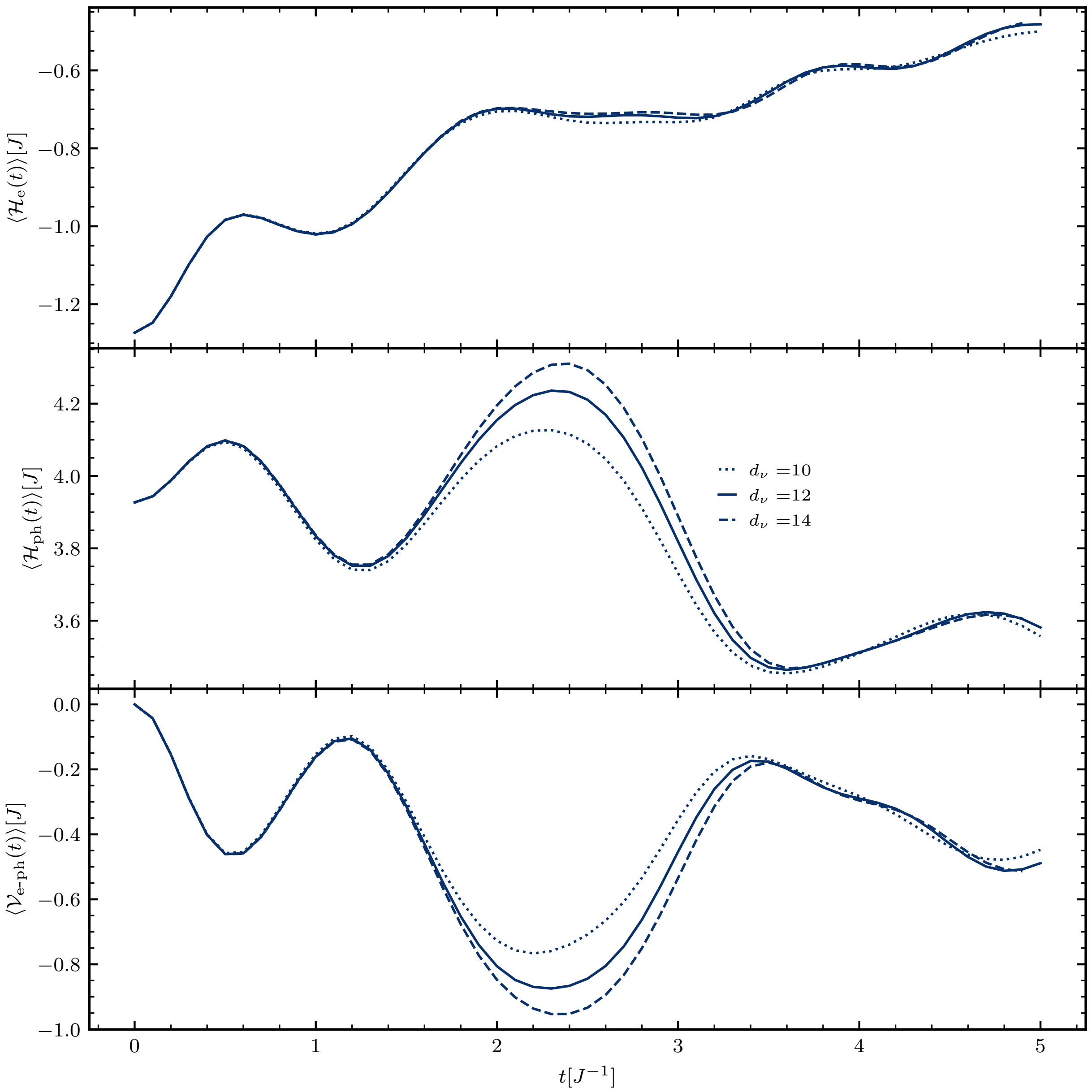

Supplementary Fig.6. **Convergence of time-evolved energy densities $\langle \mathcal{H}_{\mathrm{e}}(t)\rangle$, $\langle \mathcal{H}_{\mathrm{ph}}(t)\rangle$ and $\langle \mathcal{V}_{\text{e-ph}}(t)\rangle$ with respect to the local phonon Hilbert space dimension d_ν used in iTEBD simulations.** We use $\epsilon_{\mathrm{TEBD}} = 10^{-3.5}$ in the simulation performed here for $g_q = 0.25$ and $\omega = \pi/2$. We find that $d_\nu = 12$ suffices to achieve convergence within a reasonable bound at all accessible times.

2. Details of propagation using direct Krylov subspace methods

We perform exact time evolution via direct Krylov space methods for system sizes $L = 3-6$ with a twisted boundary condition: $e^{i(\pi/2)L}$, employing a parallelization with respect to the local bosonic parity sectors. For small system sizes, convergence with respect to the local bosonic Hilbert space dimension can be achieved, while for $L = 6$ we are restricted to a truncated bosonic Hilbert space dimension $d_\nu = 8, 10$.

B. Details of simulations of the effective model

We simulate the time evolution of the initial state $|0\rangle$ in the squeezed basis under the action of $\mathcal{H}_{\text{eff.}}$ using iTEBD, employing $d_\nu = 12$ phonon states to accurately represent the initial coherent state. We use a fourth-order trotterization scheme for the iTEBD time evolution with time-steps $\mathrm{d}t$. After each time-step, we truncate the Schmidt values of a two-site unit cell state embedded in an infinite system. We ensure that the bond dimension χ of the time-evolved state after each time-step does not saturate an upper bound of 5000. We converge our results with respect to both $\mathrm{d}t$ and ϵ_{TEBD}, finding that $\mathrm{d}t = 0.1$ and $\epsilon_{\text{TEBD}} = 10^{-3.5}$ provide satisfactory convergence and access to timescales $t \sim 5J^{-1}$ for the largest coupling ($g_q = 0.25$) and smallest phonon frequency ($\omega = \pi/2$) considered.
